# Androgen up-regulation of Twist1 gene expression is mediated by ETV1

**DOI:** 10.7717/peerj.8921

**Published:** 2020-04-09

**Authors:** Prabesh Khatiwada, Archana Kannan, Mamata Malla, Megan Dreier, Lirim Shemshedini

**Affiliations:** Department of Biological Sciences, University of Toledo, Toledo, OH, USA

**Keywords:** Androgen, Androgen receptor, Twist1, ETV1, Metastasis, Prostate cancer

## Abstract

Twist1, a basic helix-loop-helix transcription factor that regulates a number of genes involved in epithelial-to-mesenchymal transition (EMT), is upregulated in prostate cancer. Androgen regulation of Twist1 has been reported in a previous study. However, the mechanism of androgen regulation of the Twist1 gene is not understood because the Twist1 promoter lacks androgen receptor (AR)-responsive elements. Previous studies have shown that the Twist1 promoter has putative binding sites for PEA3 subfamily of ETS transcription factors. Our lab has previously identified Ets Variant 1 (ETV1), a member of the PEA3 subfamily, as a novel androgen-regulated gene that is involved in prostate cancer cell invasion through unknown mechanism. In view of these data, we hypothesized that androgen-activated AR upregulates Twist1 gene expression via ETV1. Our data confirmed the published work that androgen positively regulates Twist1 gene expression and further showed that this positive effect was directed at the Twist1 promoter. The positive effect of androgen on Twist1 gene expression was abrogated upon disruption of AR expression by siRNA or of AR activity by Casodex. More importantly, our data show that disruption of ETV1 leads to significant decrease in both androgen-mediated upregulation as well as basal level of Twist1, which we are able to rescue upon re-expression of ETV1. Indeed, we are able to show that ETV1 mediates the androgen upregulation of Twist1 by acting on the proximal region of Twist1 promoter. Additionally, our data show that Twist1 regulates prostate cancer cell invasion and EMT, providing a possible mechanism by which ETV1 mediates prostate cancer cell invasion. In conclusion, in this study we report Twist1 as an indirect target of AR and androgen regulation through ETV1.

## Introduction

Androgen signaling via the Androgen Receptor (AR) drives the development and progression of prostate cancer (Balk, 2002; Jenster, 1999). AR, a member of the nuclear receptor superfamily, is a ligand-dependent transcription factor that regulates the transcription of genes involved in prostate tumor growth and survival (Mangelsdorf et al., 1995). Therefore, the primary course of treatment for metastatic prostate cancer is to inhibit AR transcriptional activity by using a combination of competitive AR antagonists and drugs that block androgen production (Laufer et al., 2000). This is known as androgen deprivation therapy (ADT). Although the initial clinical response to ADT is excellent, most tumors relapse within 2 years (Feldman & Feldman, 2001). Tumor recurrence indicates the development of a lethal castration-resistant prostate cancer (CRPC) (Yuan & Balk, 2009), against which the second-generation anti-androgen Enzalutamide was developed (Tran et al., 2009). While this drug is effective against CRPC, resistance often develops and, when this happens, the disease outcome is death (Joseph et al., 2013; Watson, Arora & Sawyers, 2015).

Castration-resistant tumors can grow and survive in the absence of androgen; however, AR expression and function are maintained in a majority of castration-resistant tumors (Kasper & Cookson, 2006; Yuan & Balk, 2009). In castration-resistant tumors, AR acquires the capacity to drive the proliferation of cells in the absence of androgen, suggesting that AR function is essential even in advanced prostate cancer (Yuan & Balk, 2009). Given the dependence of prostate cancer on AR, it is essential that we identify AR-regulated genes that are involved in prostate tumor metastasis. Understanding how AR-regulated genes promote tumor metastasis could be beneficial for the development of drugs that target critical AR-regulated signaling pathways in lethal metastatic prostate cancer. One important gene involved in metastasis is Twist1, which was identified several years ago to be androgen-regulated in prostate cancer (Eide et al., 2013).

Twist1 has multiple pro-cancer roles such as tumor initiation, cell proliferation, development of resistance to anti-cancer drugs, and inhibition of apoptosis. However, the most well characterized role of Twist1 is in cell invasion, epithelial-to-mesenchymal transition (EMT), and tumor metastasis (Garg, 2013; Kang & Massagué, 2004; Karreth & Tuveson, 2004; Yang et al., 2004). Twist1 induces malignant transformations in melanoma (Hoek et al., 2004), lung adenocarcinoma (Pallier et al., 2012), gastric carcinomas (Sung et al., 2011; Valdés-Mora et al., 2009) and T-cell lymphomas (Van Doorn et al., 2004). Twist1 upregulation has been implicated in bone metastasis of prostate cancer and high Twist1 expression is indicative of poor prognosis (Yuen et al., 2008). Twist1 appears to induce EMT in prostate cancer cell lines by repressing E-Cadherin expression (Kwok et al., 2005). Twist1 expression is increased in malignant as compared to non-malignant prostate tissues and prostate tumors with high Gleason grades exhibit high Twist1 protein levels (Gajula et al., 2013). In addition, Twist1 knockdown in androgen-independent prostate cancer cell lines results in decreased cell invasiveness (Kwok et al., 2005).

Although high Twist1 expression is correlated with high-grade metastatic prostate tumors, there is no evidence to suggest that Twist1 is causally involved in prostate tumor cell invasion. The mechanisms that trigger aberrant expression and up-regulation of Twist1 in prostate cancer are also poorly understood. As mentioned above, Eide et al. (2013) published that Twist1 is androgen-regulated in prostate cancer, but they failed to provide a mechanism. Based on the failure to demonstrate AR recruitment to the Twist1 promoter (Wang et al., 2009), we hypothesized that AR indirectly targets the Twist1 promoter. In this study, we show that Ets Variant 1 (ETV1), which is a direct target of AR (Cai et al., 2007b), is able to activate the Twist1 promoter through a specific proximal region of the promoter and ETV1 is necessary for androgen up-regulation of Twist1 gene expression. We further demonstrate that ETV1 activity on the Twist1 promoter is differentially affected by transcriptional coactivators, with both positive and negative effects depending on the coactivator. Finally, siRNA depletion of Twist1 in prostate cancer cells caused increased E-Cadherin expression and decreased N-Cadherin expression and the opposite effects on cell migration, mimicking what was observed with AR depletion, suggesting that the AR effect on EMT and migration are mediated by Twist1. This contention is supported by our finding that Twist1 depletion significantly comprised androgen induction of cell migration. Collectively, these data suggest that AR induces expression of Twist1 via ETV1, leading to enhanced EMT and migration of prostate cancer cells.

## Materials and Methods

### Cell culture and androgen treatment

LNCaP, CWR-22Rv1 (Sramkoski et al., 1999), PC3 and C81 (Igawa et al., 2002) cells of low passage (passage 7–30) were cultured in RPMI-1640 (Corning; Cat# MT10041CV) containing 10% Fetal Bovine serum (FBS) (Gibco, Waltham, MA, USA), Penicillin-Streptomycin (Corning, New York, NY, USA) and L-Glutamine (Corning, New York, NY, USA). For androgen treatment, cells were grown in RPMI-1640 (Cat# MT17105CV; Corning, New York, NY, USA) containing 2% FBS extracted with dextran-coated charcoal (DCC) (Hyclone, Logan, UT, USA) and ethanol (−), or 10 nM R1881 (+), a synthetic androgen, was added 48 h later; 10 nM R1881 dissolved in Ethanol has previously been shown by our lab and others to activate AR in prostate cancer cells (Cai et al., 2007a; Jenster, 1999). For, Casodex treatment, 50 nM Casodex (Cayman Chemicals, Ann Arbor, MI, USA) was added to the cells after 48 h in 2% DCC. HEK 293 cells, grown in DMEM (Cat# MT10013CV; Corning, New York, NY, USA) medium containing 10% FBS, Penicillin-Streptomycin and l-Glutamine, were used for luciferase assay. LNCaP, PC3, 22RV1 and HEK 293 cells were purchased from American Type Culture Collection (ATCC^®^, Manassas, VA, USA), while C81 cells were kindly gifted by Dr. Ming-Fong Lin (University of Nebraska, Omaha, NE, USA).

### siRNA transfection and rescue

AR (Cat# HSS100619; Thermofisher, Waltham, MA, USA), Twist1 (Cat# L-006434), ETV1 (Cat# J-003801-07) and control (Cat# D-001810) (Dharmacon RNAi Technology, Lafayette, CO, USA) siRNA were transfected at 50 µM final concentration using Lipofectamine RNAiMAX reagent (Invitrogen, Carlsbad, CA, USA) following the manufacturer’s protocol. Additionally, for shRNA knockdown, cells were infected with lentivirus (pLKO.1 backbone) expressing shRNA for AR (CGTGCAGCCTATTGCGAG), ETV1 (CAATGTCAGTGCCTATGAT) or scramble in presence of 10 µg/ml Polybrene (Millipore, Burlington, MA, USA). To make the lentivirus expressing shRNA, HEK 293T cells were transfected with pSPAX2 (packaging plasmid), pMD2.G (Envelope plasmid) and plKO.1 (viral backbone) plasmid expressing shRNA, and viruses were collected 72 h post transfection. For ETV1 rescue experiment, 24 h post lentiviral (shRNA) infection, LNCaP cells were transfected with ETV1 (ETV1/pTL1) or empty vector (/pTL1) plasmids using Lipofectamine^®^ 3000 (Invitrogen, Carlsbad, CA, USA) transfection reagent as indicated.

### Plasmid and reporter gene assay

The Twist1 Luciferase reporter plasmid (Twist1-Luc) and it’s mutant forms (Mut-A, Mut-e and Mut-A&E) were kindly obtained from Dr. Jianming Xu, Baylor College of Medicine, Houston, TX and has been described in their previously published report (Xu et al., 2014). Additionally, pSG5-ARE-Luc (Androgen response element) and pGL3-Luc (empty) were used in this study and has been described previously (Cai, Hsieh & Shemshedini, 2007). For reporter gene assay, cells were transfected with 0.5 µg of the reporter plasmid along with 0.5 µg pCH110 (which express β-galactosidase and used as control for transfection efficiency), using Lipofectamine^®^ 3000 (Invitrogen, Carlsbad, CA, USA) following manufacturers protocol. Luciferase assays were performed in triplicates 48 h post-transfection, as previously described (Chen et al., 2006). All Luciferase values are representing the average of three independent experiments plus standard deviations.

### RNA isolation and quantitative RT-PCR analyses

RNA isolation was performed using the Trizol reagent (Invitrogen, Carlsbad, CA, USA) following manufacturer’s instructions and quantitative real time PCR (qRT-PCR) was performed using iTaq Universal SYBR^®^ Green Supermix (Bio-Rad, Hercules, CA, USA). The PCR primers were purchased from IDT Technologies and the upstream and downstream primers, respectively, used for each gene were: AR, 5′-GCATGGCAGAGTGCCCTATC-3′ and 5′ TCCCAGAGTCATCCCTGCTTCAT-3′; Twist1, 5′-GTCCGCAGTCTTACGAGGAG-3′ and 5′-CCAGCTTGAGGGTCTGAATC-3′; ETV1, 5′-TACCCCATGGACCACAGATT-3′ and 5′-CACTGGGTCGTGGTACTCCT-3′; PSA, 5′-GCAGCATTGAACCAGAGGAG-3′ and 5′-CCCATGACGTGATACCTTGA-3′; E-Cadherin, 5′-TGCCCCAGAAAATGAA AAAGG-3′ and 5′-GTGTATGTGGCAATGCGTTC-3′; N-Cadherin, 5′-ATTGTGGGTG CGGGGCTTGG-3′ and 5′-GGGTGTGGGGCTGCAGATCG-3′; and GAPDH, 5′-CGAC CACTTTGTCAAGCTCA-3′ and 5′-AGGGGAGATTCAGTGTGGTG-3′. GAPDH was used as control for mRNA amounts. qRT-PCR measurements were quantified following the 2^−ΔΔCt^ method (Livak method) and are given relative to GAPDH expression and are representing the average of three replicates plus standard deviations.

### Chromatin immunoprecipitation

LNCaP cells grown in 2% DCC for 48 h were treated with ethanol (−) or R1881 (+) for 24 h. The cells were fixed with 0.75% formaldehyde for 15 min and quenched with 0.125 M glycine. Cells were washed with PBS twice and lysed with ChIP lysis buffer to isolate the chromatin. Lysates were sonicated (12 cycles of 15 s ON and 45 s OFF and 18% power) and DNA was sheared to an average length of 300–500 bp. Genomic DNA (Input) was prepared by treating aliquots of chromatin with RNase, proteinase K, and heat for de-crosslinking, followed by purification. The purified DNA was quantified on a Nanodrop spectrophotometer. A total of 100 µg of DNA was used for each IP following dilution in RIPA buffer. The protein A/G beads (Santa-Cruz, CA, USA) were precleaned with RIPA buffer twice, blocked with salmon sperm, washed, and resuspended in RIPA buffer (1:1), after which 60 µg was used for each IPs. ChIP was performed using anti-AR (Cat# 5153; Cell Signaling Technology, Danvers, MA, USA), anti-ETV1 (Cat# LS‑C352170; LS Bio, Seattle, WA, USA) and anti-IgG (Cat# sc-2025; Santa-Cruz, CA, USA) control. After overnight incubation, the Protein A/G Agarose-Immune complex was washed, eluted, reverse-crosslinked and purified. ChIP-qRT PCR was performed using primers for the A 5′-TCCCCTCCCCTTCCCAAATT-3′ and 5′-TCAAAAATAAATAAAGAGAT-3′, E 5′-ATGGGGCTGCCACCGCGG-3′ and 5′-TGGGGGCAGCAGTGTCATT-3′, E1 5′-ATGGGGCTGCCACCGCGG-3′ and 5′-CCCTCCACCCGCCCTCCCTA-3′ and E2 5′-CGTTTTTGAATGGTTTGGGA-3′ and 5′-TGGGGGCAGCAGTGTCATT-3′ (two halves of the E) regions of the Twist1 promoter and the PSA (5′-GACAACTTGCAAACCTGCTC-3′ and 5′-GATCCAGGCTTGCTTAC TGT-3′) enhancer region. ChIP values are represented as fold enrichment relative 2% of input. The fold values are represented relative to the IgG control (anti-IgG ChIP, no hormone). Each value is the average of three repeats plus standard deviation.

### Western blotting

Cells were lysed using M-PER^®^ (Thermo-Scientific, Waltham, MA, USA) reagent supplemented with 1 mM Sodium-orthovanadate (Na_3_VO_4_), 1 mM phenylmethylsulfonyl fluoride (PMSF) and Pierce™ Protease Inhibitor Tablets (Thermo-Scientific, Waltham, MA, USA) following manufacturer’s instructions; centrifuged at 14,000×*g* for 10 min at 4 °C. The supernatant (cell lysate) was boiled for 5 min in SDS-PAGE sample buffer (250 mM Tris-HCl, pH 6.8, 10% sodium dodecyl sulfate, 10% β-mercaptoethanol, 40% glycerol, 0.01% brom0phenol blue). The solubilized proteins were separated by SDS–PAGE followed by Western blotting with the indicated antibodies and visualization with the enhanced chemiluminescence detection system. The antibodies against AR (Cat# sc-7305, AR-441; Santa-Cruz, CA, USA), Twist1 (Cat# 46702; Cell Signaling Technology, Danvers, MA, USA), ETV1 (Cat# LS‑C352170; LS Bio, Seattle, WA, USA), N-Cadherin (Cat# sc-7939; Santa-Cruz, CA, USA), E-Cadherin (Cat# 3195; Cell Signaling Technology, Danvers, MA, USA) and β-actin (Cat# MA5-15739; Invitrogen, Carlsbad, CA, USA), were used in this study. Additionally, HRP-linked Anti-rabbit IgG (#7074; Cell Signaling Technology, Danvers, MA, USA) or Anti-mouse IgG (#7076; Cell Signaling Technology, Danvers, MA, USA) were used as secondary antibodies. β-actin was used as loading control. Each Western blot image shown in figures is a representative figure of three experiments. The Western blots were quantified using ImageJ (NCBI) and shown in bar graph as average relative band intensity (normalized to β-actin) plus standard deviation of three independent experiments.

### Cell migration assay

Cell migration was measured using the Cytoselect™ 24-Well Cell Invasion Assay kit, 8 µm (fluorometric quantitation) (Cell Biolabs, San Diego, CA, USA) following the manufacturer’s protocol. Briefly, cell suspension containing 100,000 cells/ml (in full serum or in serum-free medium treated with or without R1881) were used to monitor cell migration into the lower chamber of the cell migration assay kit, which contained RPMI 1640 medium with 10% DCC-stripped serum. After 48 h of incubation at 37 °C, cells were quantified by fluorescence using a microplate reader. For each experiment, cells for every conditions were grown in triplicates on a 48-well plate to measure cell proliferation using MTT assay (Morgan, 1998), using which cell migration was standardized. The cell migration value (folds) are normalized to control, and they represent the average of three independent experiments plus standard deviation.

### Statistical analysis

All experiments were done at least three times and data are presented in bar graph as averages plus standard deviations. The Student’s *T*-test was performed to compare the difference between any pair of data and calculate *p*-values. The statistical significance is indicated by the asterisks (*p ≤ 0.05, ***p ≤ 0.01).

## Results

### Androgen treatment induces Twist1 expression in androgen-dependent and androgen-independent prostate cancer cell lines

**Figure 1:**
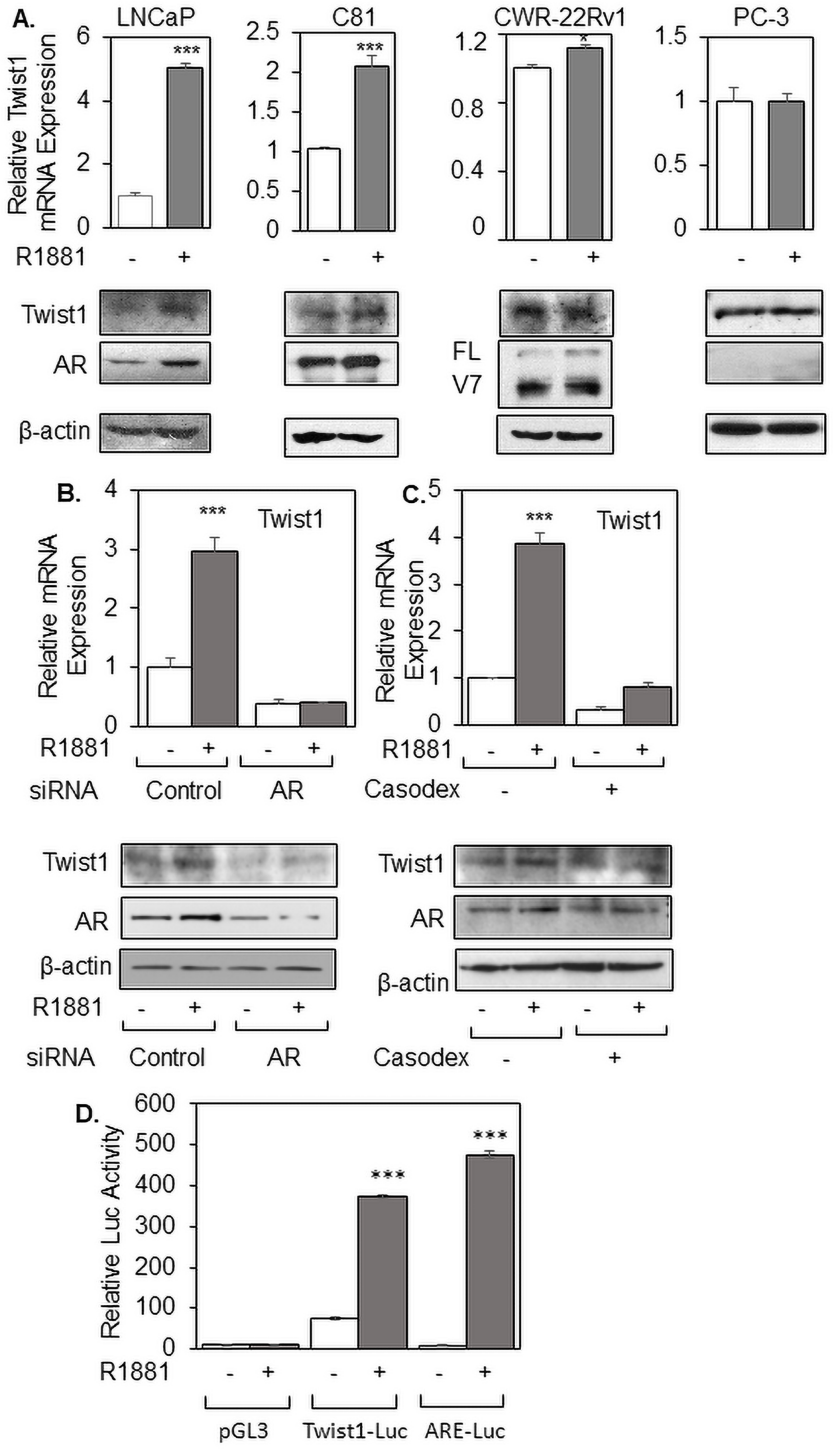
Androgen induces Twist1 expression in prostate cancer cells and AR is required for this induction. (A) LNCaP, C81, CWR-22Rv1 and PC3 cells or (B–D) LNCaP cells were grown in 2% DCC-serum with ethanol (−) or 10 nM R1881 (+) for 48 h with (B) transfection of control or AR siRNA, (C) treatment with or without 50 nM Casodex, or (D) transfection with pGL3, Twist1-Luc, or ARE-Luc. (A–C) Twist1 mRNA was measured by qRT-PCR and Twist1 and AR proteins were measured by Western blotting. Note that β-actin was used as a loading control for the Western blots. (D) Luciferase reporter activity was measured standardized to pCH110 (control transfection). The Luciferase activity shown is relative to the activity of pGL3 (−), which was set as 1. Bar graphs represent averages of three independent experiments plus standard deviation. The Student’s *T*-test was performed to show statistical significance (*p < 0.05, ***p < 0.01), as indicated by the asterisks.

Our lab has previously used Affymetrix microarray analysis to identify several androgen-regulated genes, including Soluble Guanyl Cyclase α1 (sGCα1), ETV1, and Multi-Drug Resistant Protein 4 (MRP4) which have been shown to have important pro-cancer roles in prostate cancer cells (Cai et al., 2007a, 2007b, 2006; Cai, Hsieh & Shemshedini, 2007). In this current study, we describe Twist1 as another androgen-regulated gene that came from the microarray analysis. As shown in Fig. 1A, endogenous Twist1 mRNA expression and protein were significantly induced by R1881 treatment in LNCaP cells, consistent with an earlier study reporting androgen regulation of Twist1 (Eide et al., 2013); LNCaP cells express AR and exhibit androgen-dependent cell growth (Horoszewicz et al., 1983). As expected (Jenster, 1999), androgen caused upregulation of AR in LNCaP cells (Fig. 1A). To determine what happens to Twist1 expression in hormone-independent cells, we used C81 cells (Igawa et al., 2002), which were derived from LNCaP cells. Interestingly, we observed reduced but significant androgen upregulation of Twist1 in the hormone-independent C81 cells (Fig. 1A), while another hormone-independent cell line, CWR-22Rv1 (Sramkoski et al., 1999), expressed high levels of Twist1 mRNA and protein unaffected by hormone (Fig. 1A). These results show that androgen regulation of Twist1 differs in different prostate cancer cells and those cells that have the same lineage (LNCaP and C81 cells) exhibit hormone-inducible expression of Twist1. High Twist1 expression was also observed in PC-3 cells, which lack AR expression (Fig. 1A). The relative band intensities of AR, AR-V7 (CWR-22Rv1) and Twist1 proteins in LNCaP (Fig. S1A), C81 (Fig. S1B), CWR-22Rv1 (Fig. S1C) and PC3 (Fig. S1D) cells were quantified using ImageJ (NCBI) software (Fig. S1). To demonstrate that androgen activation of Twist1 expression is dependent on AR, we knocked down AR by siRNA (Fig. 1B; Fig. S2A) or treatment with the anti-androgen Casodex (Fig. 1C; Fig. S2B). Treatment with either siRNA (Fig. 1B) or Casodex (Fig. 1C) strongly inhibited androgen upregulation of Twist1 expression at both the mRNA and protein levels. The relative band intensity of AR and Twist1 protein expression were quantified for both AR knockdown (Fig. S2C) and Casodex treatment (Fig. S2D). As expected, Casodex treatment also blocked androgen induction of the classical AR target gene ETV1 (Fig. S2B). Depletion of AR by siRNA also led to significantly decreased expression of Twist1 mRNA in the two hormone-independent cell lines C81 (Fig. S3A) and CWR-22Rv1 (Fig. S3B), showing that endogenous AR is required for the hormone-independent expression of Twist1 mRNA in these cells. To determine if the androgen upregulation of Twist1 occurs at transcriptional level, we used a Luciferase reporter gene driven by the Twist1 promoter (Himes & Shannon, 2000; Qin et al., 2009; Xu et al., 2014). As shown in Fig. 1D, R1881 strongly activated the Twist1-Luc reporter gene to a level comparable to the positive control ARE-Luc, while the promoter-less pGL3 yielded weak activity unresponsive to androgen.

### Androgen regulation of Twist1 is indirect and is mediated by ETV1

Activation of Twist1 promoter upon androgen treatment suggested that AR-regulation of Twist1 is transcriptional. However, analysis of whole genome AR ChIP-on-ChIP data in LNCaP cells from a previous study (Wang et al., 2009) suggested that AR is not recruited to the Twist1 promoter. Additionally, CHIP-Seq analysis of the Twist1 promoter in LNCaP cells showed that there is no AR binding to this promoter (GEO Dataset GSE32345). These findings suggest that androgen regulation of Twist1 is indirect and may be mediated by an androgen-regulated transcription factor(s).

**Figure 2:**
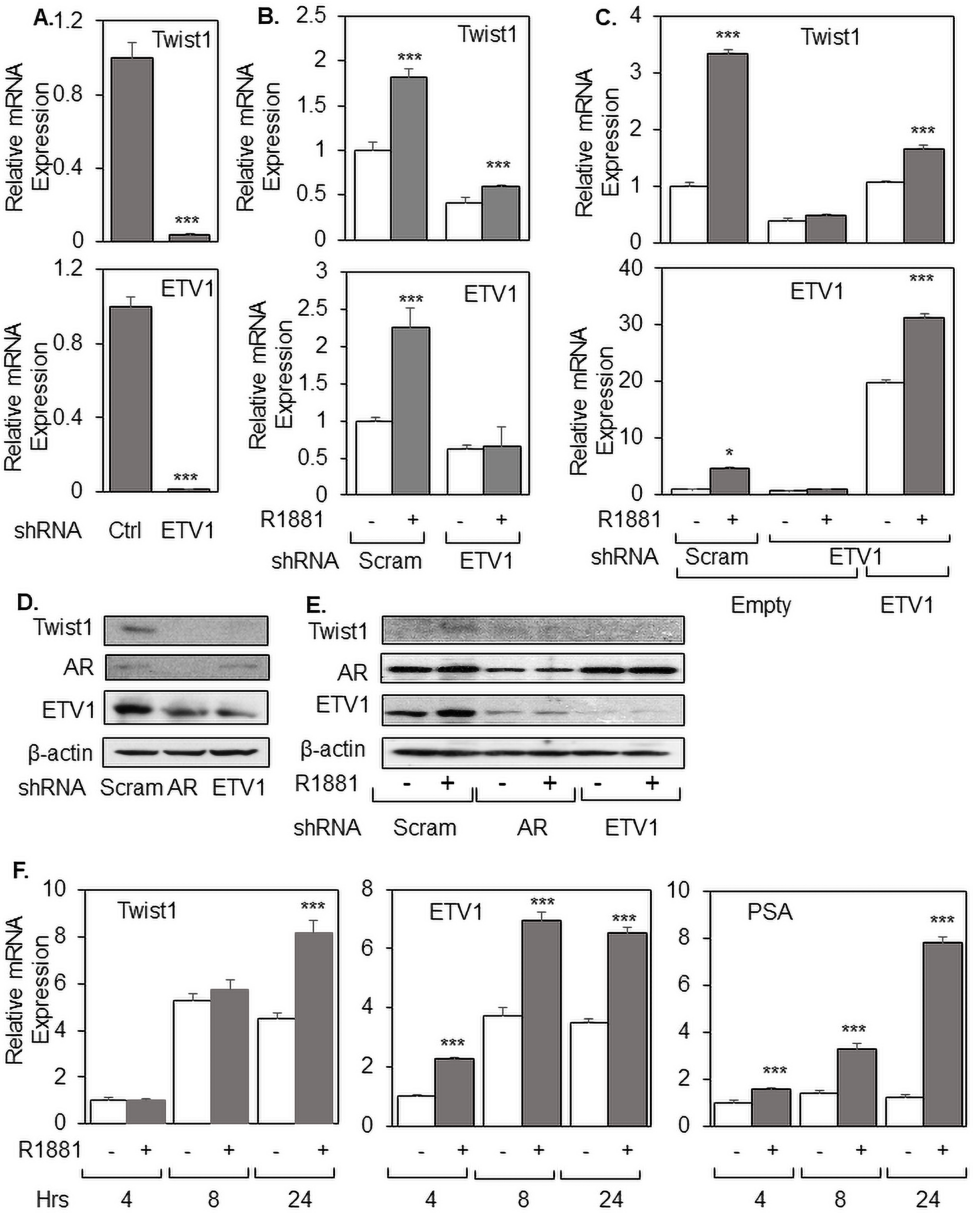
ETV1 is required for androgen-induced expression of Twist1. LNCaP cells grown in full serum (A) or treated with ethanol (−) or R1881 (+) (B and C) were infected with Lentivirus expressing scramble or ETV1 shRNA. In (C), ETV1 rescue was performed by transfecting Empty or ETV1 expression plasmid using Lipofectamine^®^ 3000. Expression of Twist1 and ETV1 were measured using qRT-PCR. Similarly, LNCaP cells grown in full serum (D) or treated with ethanol (−) and R1881 (+) (E) were infected with lentivirus expressing Scramble, AR and ETV1 shRNA and expression of Twist1, ETV1, AR and β-actin were measured using Western blots. (F) LNCaP cells grown in 2% DCC for 48 h were treated with ethanol (−) or R1881 (+) for 4, 8 or 24 h and expression of Twist1, ETV1 and PSA was measured using qRT-PCR. Bar graphs represent averages of three independent experiments plus standard deviations. The Student’s *T*-test was performed to show statistical significance (*p < 0.05, ***p < 0.01), as indicated by the asterisks.

While the cloned 2.8 kb Twist1 promoter lacks AREs, it contains by sequence multiple binding regions for ETS transcription factors. Qin et al. (2009) have previously shown that Polyoma Enhancer Protein 3 (PEA3), an ETS transcription factor, directly activates the Twist1 promoter. PEA3 (also known as Ets Variant 4 (ETV4)) belongs to PEA3 family of ETS transcription factors with other members being ETV1, Ets Variant 2 (ETV2) and Ets Variant 5 (ETV5). Our lab has previously published that ETV1 is androgen-regulated gene in prostate cancer cells, while other PEA3 family genes are poorly expressed and not under androgen regulation (Cai et al., 2007b). Based on these data, we hypothesized that ETV1 mediates androgen-dependent upregulation of Twist1. To study a possible role for ETV1 in Twist1 expression in prostate cancer (PCa) cell, we infected LNCaP cells grown in full serum with a lentivirus expressing ETV1 shRNA, which interestingly caused a decrease in endogenous Twist1 mRNA expression, as measured by qRT-PCR (Fig. 2A); a similar reduction in Twist1 protein expression was observed with either AR or ETV1 shRNA (Fig. 2D, quantified in Fig. S4A). To directly test our hypothesis that ETV1 mediates androgen upregulation of Twist1, we repeated the previous experiment using medium containing 2% DCC instead of full serum in order to measure the effect of androgen. Depletion of ETV1 by shRNA almost completely abolished the androgen upregulation of Twist1 mRNA (Fig. 2B) and protein (Fig. 2E, quantified in Fig. S4B), strongly suggesting that endogenous ETV1 is required for androgen induction of Twist1 expression; ETV1 depletion also reduced by about 50% Twist1 mRNA expression in the absence of R1881 (Fig. 2B). To verify our finding that reduced Twist1 expression is indeed due to shRNA depletion of endogenous ETV1, we attempted a rescue experiment by transiently over-expressing ETV1 (Fig. 2C). As expected, androgen treatment enhanced Twist1 mRNA expression by about three-fold which was completely abolished upon ETV1 knockdown (Fig. 2C). Transient over-expression of ETV1 (ETV1/pTL1) following shRNA mediated ETV1 knockdown partially and significantly rescued Twist1 mRNA in both the absence and presence of R1881 (Fig. 2C). Collectively, these data strongly argue that ETV1 regulates basal Twist1 expression and, more importantly, mediates the androgen-dependent expression of Twist1 in prostate cancer cells.

If, as our data suggest, Twist1 is an indirect target of AR and its androgen upregulation is mediated by ETV1, we should observe different kinetics in androgen upregulation of Twist1 and ETV1. Indeed, androgen induction of Twist1 occurred sometime between 8 and 24 h (Fig. 2F). By contrast, ETV1 and PSA, both of which are direct targets of AR (Cai et al., 2007b), exhibited androgen induction within 4 h (Fig. 2F). These data clearly demonstrate that androgen induction of ETV1 occurs before induction of Twist1, consistent with our contention that ETV1 mediates the androgen activation of Twist1 expression.

### ETV1 directly activates the Twist1 promoter

**Figure 3:**
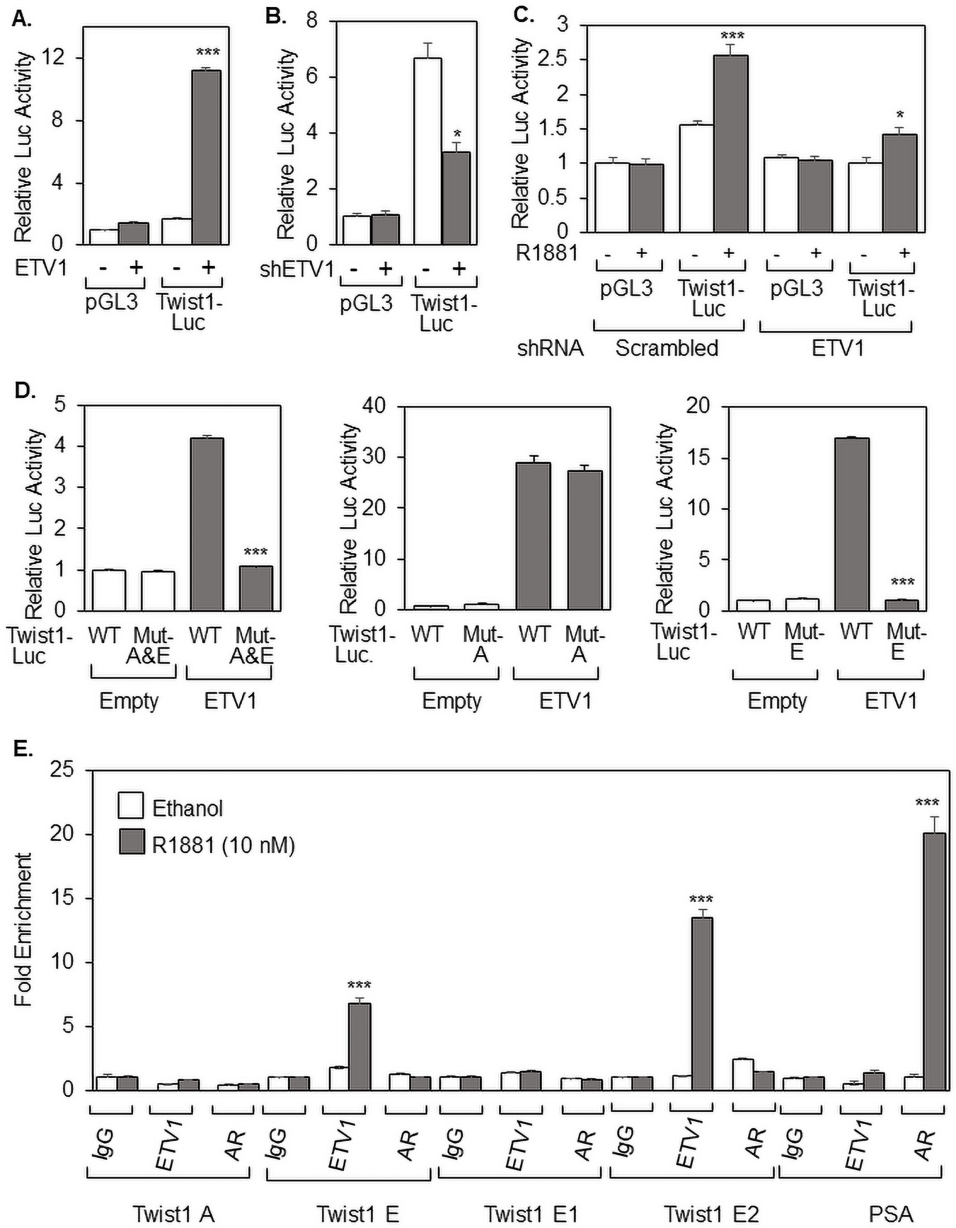
ETV1 activates Twist1 expression through a proximal Ets-responsive region of the Twist1 promoter. (A and D) HEK cells were co-transfected with pGL3, Twist1-Luc (called WT in D), or Twist1-Luc reporter plasmids mutated for different regions (A, E, or A and E) and ETV1 expression plasmid (+) or empty plasmid (−), as indicated. (B and C) LNCaP cells, grown in either (B) full serum or (C) 2% DCC-serum and treated with ethanol (−) or 10 nM R1881 (+), were co-transfected with pGL3 or Twist1-Luc and scrambled shRNA (−), ETV1 shRNA (+), or empty vector (−), as indicated. The Luciferase activity shown is relative to the activity of pGL3 (−), which was set as 1. (E) ChIP assay was performed with LNCaP cells grown in 2% DCC-serum with ethanol or 10 nM R1881, as shown, to measure recruitment of ETV1 or AR to the Twist1 promoter regions A, E, E1, or E2 and PSA. ChIP values are represented as fold enrichment relative to the IgG control (anti-IgG ChIP, no hormone), which were first standardized to Input. Bar graphs represent averages of three independent experiments plus standard deviations. The Student’s *T*-test was performed to show statistical significance (*p < 0.05, ***p < 0.01), as indicated by the asterisks.

To determine if ETV1 directly targets the Twist1 promoter, we used the same Twist1-Luc reporter plasmid that was used in Fig. 1D. As shown in Fig. 3A, exogenous ETV1 had a strong positive effect on the Twist1-Luc reporter in HEK cells, while there was no effect on the promoter-less pGL3 plasmid, paralleling what was observed earlier with androgen regulation of the Twist1 promoter (see Fig. 1D). Since LNCaP cells express endogenous ETV1 (see Fig. 2D), we used shRNA to knockdown this endogenous ETV1, resulting in a significant reduction in Twist1-Luc reporter activity (Fig. 3B). ETV1 knockdown by shRNA also abolished androgen activation of the Twist1 promoter, but it had no effect on the promoter-less pGL3 (Fig. 3C), suggesting that endogenous ETV1 mediates androgen activation of the Twist1 promoter and supporting our earlier finding (see Fig. 2C) that endogenous ETV1 mediates androgen upregulation of Twist1 gene expression. These data clearly show that both exogenous and endogenous ETV1 can activate the Twist1 promoter. Qin et al. (2009) generated truncations of the Twist1 promoter to identify the region targeted by PEA3, and we used the same Twist1 promoter (2.8 kb) truncations to study ETV1 activity. Deletion of both promoter Regions A (201–331 bp) and E (2,085–2,420 bp) resulted in abolishment of ETV1 activity (Fig. 3D). Interestingly, deletion of Region A had no effect, while deletion of Region E almost completely eliminated ETV1 activation of the Twist1 promoter (Fig. 3D), mimicking what was observed with deletion of both promoter regions and strongly suggesting that ETV1 activity on the Twist1 promoter is mediated via Region E. To determine if ETV1 directly acts on the Twist1 promoter, we performed chromatin immunoprecipitation (ChIP) and determined that ETV1 is recruited to the Twist1 Region E and this is significantly stimulated by androgen, while there was not significant recruitment of ETV1 to Region A (Fig. 3E), entirely consistent with our Luciferase data (see Fig. 3D). Interestingly, we were able to further map the ETV1 recruitment site to the downstream half of Region E which we call E2 (2,274–2,420 bp), which exhibited markedly stronger ETV1 binding than Region E in either the absence or presence of androgen (Fig. 3E). Importantly, there was no measurable AR recruitment to either Region A or E, while strong hormone-inducible AR binding was measured to the PSA promoter as a positive control (Fig. 3E). Collectively, these data show recruitment of ETV1, but not AR, to the Twist1 promoter, arguing for a direct transcriptional effect of ETV1 on Twist1 gene expression.

As a transcriptional activator, ETV1 transcriptional activity is dependent on coactivators, and thus we tested several coactivators for their effects on ETV1-induced activation of the Twist1 promoter. Interestingly, the two closely related proteins Steroid Receptor Coactivator 1 (SRC-1) and Steroid Receptor Coactivator 2 (SRC-2) (Wang, Lonard & O’Malley, 2016) both enhanced ETV1 stimulation of Twist1-Luc, whereas the more distantly related protein Steroid Receptor Coactivator 3 (SRC-3) was inhibitory (Fig. S5A). CREB-Binding Protein (CBP) and p300 are very closely related coactivators with highly overlapping functions (Bedford et al., 2010), but they differ in ETV1 regulation of the Twist1 promoter, with p300 strongly inhibiting and CBP having a positive effect (Fig. S5B).

### Twist1 mediates prostate cancer cell migration

**Figure 4:**
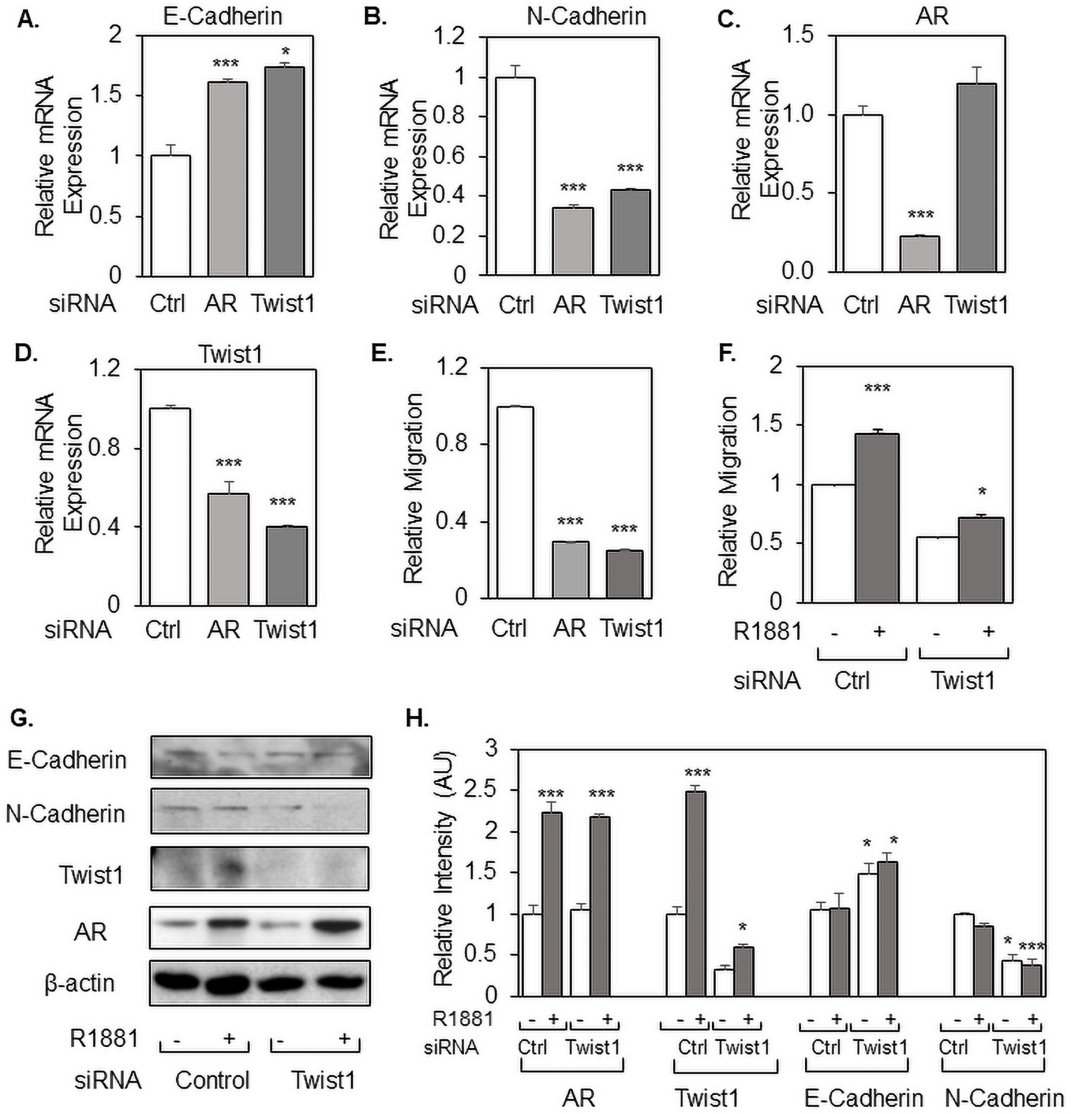
Twist1 is required for androgen-induced migration of prostate cancer cells. LNCaP cells were transfected with control (Ctrl), AR, or Twist1 siRNA and measured by qRT-PCR for mRNA expression of E-Cadherin (A), N-Cadherin (B), Twist1 (C), or AR (D). The cells were also monitored for cell migration (E and F), which was standardized to no R1881 and control siRNA, which was set as 1. The cells in (F) were measured by Western blotting for expression of E-Cadherin, N-Cadherin, Twist1 and AR (G), which was quantified using ImageJ and shown as a bar graph (H). In (F), cells were grown in 2% DCC-serum with ethanol (−) or 10 nM R1881 (+). Bar graphs represent averages of three independent experiments plus standard deviations. The Student’s *T*-test was performed to show statistical significance (*p < 0.05, ***p < 0.01), as indicated by the asterisks.

Twist1 is a transcription factor known to regulate the expression of multiple genes involved in cell migration, EMT, and tumor metastasis (Kang & Massagué, 2004; Karreth & Tuveson, 2004; Yang et al., 2004). E-Cadherin is perhaps the best characterized Twist1 target gene, and one mechanism by which Twist1 appears to induce cell migration is by repressing E-Cadherin expression (Kang & Massagué, 2004; Kwok et al., 2005). Indeed, knockdown of Twist1 in LNCaP cells resulted in a significant increase in E-Cadherin gene expression, mimicking what was observed with AR knockdown (Fig. 4A). Conversely, N-Cadherin expression was markedly decreased in response to either Twist1 or AR knockdown (Fig. 4B). Furthermore, the effect of Twist1 knockdown, in presence or absence of R1881, had similar effect on E-Cadherin and N-Cadherin protein expression (Fig. 4G, Quantified in Fig. 4H). Importantly, siRNA knockdown of AR reduced Twist1 gene expression as well as did Twist1 siRNA (Fig. 4C), while Twist1 knockdown had no effect on AR expression (Fig. 4D). Consistent with these gene expression changes, Twist1 depletion strongly repressed the migration of prostate cancer cells, comparable in effect to AR knockdown (Fig. 4E). Knockdown of either AR or Twist1 did not affect the growth of prostate cancer cells for the short time of the migration assay (Fig. S6A), excluding the possibility that reduced cell number could be responsible for the reduced cell migration. As we have shown before (Cai et al., 2007b), R1881 significantly increased the migration of LNCaP cells and this increased migration was completely abolished when Twist1 was siRNA depleted (Fig. 4F), suggesting that Twist1 mediates prostate cancer cell migration; there was no effect on cell proliferation (Fig. S6B).

## Discussion

AR is essential for the initiation and progression of prostate cancer. While the multiple AR pro-cancer functions have been well studied, its role in metastasis is poorly understood. Among the many AR target genes that have been discovered, only a few are known to be involved in metastasis. Perhaps the best example thus far is SNAI2, encoding the transcription factor Slug that is directly associated with the invasive potential of prostate cancer cells (Vuong et al., 2016). Another target of AR, that was originally identified by Eide et al. (2013) as a novel androgen-regulated gene, is Twist1, which forms the basis of the study presented here.

In that earlier report of AR up-regulation of Twist1 in prostate cancer cells by Eide et al. (2013), the authors did not provide a mechanism for androgen regulation of Twist1. This may have been due, in part, to the indirect route by which AR acts on Twist1, as we have shown here. A hallmark of an indirect transcriptional effect by a transcription factor is the absence of recruitment of that factor to the gene’s promoter, and that is precisely what has been learned about AR recruitment to the Twist1 promoter. ChIP-on-ChIP analysis did not detect AR on the Twist1 promoter (Wang et al., 2009) and more recent ChIP analysis verified this finding that AR is not recruited to the Twist1 promoter (GEO GSE32345). Consistent with these data, we failed to detect in this study by ChIP analysis AR binding to the Twist1 promoter. Thus, these two findings led us to consider an indirect effect of androgen on Twist1 expression, which was significantly aided by published work of Qin et al. (2009) showing that PEA3 can indeed bind to and activate the Twist1 promoter. PEA3 is not expressed in LNCaP cells and it is not androgen-inducible (Cai et al., 2007b). Indeed, among the four members of the PEA3 family of transcription factors, only ETV1 is expressed and exhibits androgen-inducible expression in prostate cancer cells (Cai et al., 2007b). Our data here clearly demonstrate both endogenous and exogenous ETV1 can promote expression of Twist1 in prostate cancer cells, and this ETV1 positive effect was also observed on a Twist1-Luc reporter plasmid, which is a consistent with a direct effect of ETV1 on the Twist1-promoter. Further evidence for a direct effect came from our finding that mutation of a putative Ets-responsive region within the Twist1 promoter disrupted ETV1 activity, suggesting that ETV1 act through the same region of the Twist1 promoter as does PEA3 (Qin et al., 2009). We also compared the kinetics of androgen induction of direct targets, such as ETV1 and PSA, both of which were upregulated within 4 h, while Twist1 expression was induced sometime between 8 and 24 h. Finally, our strongest data for a direct effect of ETV1 on the Twist1 promoter derived from a ChIP assay showing that ETV1 binds to the same region of the Twist1 promoter that that mediates the transactivation of this promoter by ETV1. Collectively, all these data strongly suggest that androgen-activated AR promotes Twist1 gene expression by through its direct target ETV1, which can act directly activate the Twist1 promoter.

Activators like AR and ETV1 depend on co-activators for their transcriptional activities (reviewed in Manteuffel-Cymborowska (1999)). The SRC family of proteins have been shown to act on different transcriptional activators, including nuclear receptors (Wang, Lonard & O’Malley, 2016) and Ets proteins (Goel & Janknecht, 2004). Interestingly, SRC-1 and SRC-2 were both able to significantly enhance the activity of exogenous ETV1 on the Twist1 promoter, while SRC-3 had no significant effect. Our findings are consistent with previously published results showing that SRC-1 can promote ETV1 activity (Goel & Janknecht, 2004), while nothing has been previously reported on SRC-2 and SRC-3 activity on ETV1. It perhaps is expected that SRC-1 and SRC-2 would have similar activities on ETV1 since these two proteins are closely related and SRC-3 is more distantly related (Xu & Li, 2003). Since Qin et al. (2009) previously showed that SRC-1 activates Twist1 expression by interacting with the Ets protein PEA3, it is possible that SRC-1 also interacts with ETV1. CBP and p300 represent another group of closely related coactivators (Bedford et al., 2010) that have been previously shown to promote ETV1 transcriptional activity by interacting with ETV1 (Papoutsopoulou & Janknecht, 2000) and, in the case of p300, acetylating ETV1 (Goel & Janknecht, 2003, 2004). Our data show that p300 surprisingly blocked ETV1 activity on the Twist1 promoter, while CBP had no significant effect. These data suggest that the specific effects of p300 and CBP on ETV1 transcriptional activity are promoter-specific and can be either positive or negative.

Twist1 is known to induce EMT in cancer by up-regulating E-Cadherin and down-regulating N-Cadherin (reviewed in Zhao et al. (2017)), and we determined that Twist1 has the same effects in prostate cancer. Indeed, siRNA depletion of Twist1 in LNCaP cells induced E-Cadherin expression and blocked N-Cadherin. Interestingly, siRNA depletion of AR had the same effects on E-Cadherin and N-Cadherin expressions as did siRNA depletion of Twist1, suggesting that the AR effect on EMT may be mediated by it indirect up-regulation of Twist1. In support of this, prostate cancer cell migration, which results from EMT, was severely compromised to the same extent when either Twist1 or AR expression was diminished by siRNA. Furthermore, the androgen promotion of cell migration was significantly reduced when Twist1 expression was depleted, suggesting that androgen up-regulation of migration is mediated by Twist1. Twist1 promotes metastasis through multiple pathways, in addition to EMT. These include Twist1 induction of intravascular migration and extravasation (Stoletov et al., 2010), invadopodia formation (Eckert et al., 2011), and vasculogenic mimicry (VM) formation (Sun et al., 2010). It will be interesting to determine in future work if the AR indirect up-regulation of Twist1 via ETV1 is involved in these other steps of prostate cancer metastasis.

## Conclusions

In this study we demonstrate that androgen-activated AR promotes the expression of Twist1 in prostate cancer cells. Since there is no evidence for AR binding to the Twist1 promoter, we hypothesized that the AR effect on Twist1 expression is indirect and mediated by a direct target gene of AR. We provide evidence that the Ets protein ETV1, whose gene is a direct target of AR, mediates the AR positive effect on Twist1. Our data show ETV1 acts through a specific region of the Twist1 promoter to induce Twist1 gene expression. We further show that Twist1 promotes EMT and migration of prostate cancer cells, and our data suggest that Twist1 plays an important role in mediating the AR induction of EMT and cell migration. Future studies can focus on the importance of Twist1 in mediating other steps of prostate cancer metastasis, including intravascular migration and extravasation and invadopodia formation.

## Supplemental Information

10.7717/peerj.8921/supp-1 Supplemental Information 1 Quantification of androgen induction of AR and Twist1 proteins in prostate cancer cells. LNCaP (A), C81 (B), CWR-22Rv1 (C), or PC3 (D) cells were grown in 2% DCC-serum with ethanol (−) or 10 nM R1881 (+) for 48 h, Western blotting was performed and quantified using ImageJ for AR and Twist1 proteins, which are shown as bar graphs (normalized to β-actin). Bar graphs represent averages of three independent experiments plus standard deviations. The Student’s *T*-test was performed to show statistical significance (*p < 0.05, ***p < 0.01), as indicated by the asterisks.

10.7717/peerj.8921/supp-2 Supplemental Information 2 AR is required for androgen induction of Twist1 gene expression. LNCaP cells were transfected with control or AR siRNA (A and B) or treated with 50 nM Casodex (an anti-androgen) (C and D) in the presence of ethanol (−) or 10 nM R1881 (+) and expression of AR (A), or ETV1 (C) were measured using qRT-PCR. The relative band intensity of AR and Twist1 proteins were quantified using ImageJ and shown as bar graphs (normalized to β-actin) in (B) and (D). Bar graphs represent averages of three independent experiments plus standard deviations. The Student’s *T*-test was performed to show statistical significance (*p < 0.05, ***p < 0.01), as indicated by the asterisks.

10.7717/peerj.8921/supp-3 Supplemental Information 3 AR is required for androgen induction of Twist1 gene expression in different prostate cancer cells. (A) C81 or (B) CWR-22Rv1 cells grown in 2% DCC with ethanol (−) or 10 nM R1881 (+) were transfected with control or AR siRNA and relative gene expression of Twist1 and AR were measured by qRT-PCR. Bar graphs represent average of 3 independent experiments plus standard deviations. The Student’s *T*-test was performed to show statistical significance (*p < 0.05, ***p < 0.01), as indicated by the asterisks.

10.7717/peerj.8921/supp-4 Supplemental Information 4 Quantification of Western blot showing that ETV1 is required for androgen-induced expression of Twist1 proteins. LNCaP cells grown in full serum (A) or treated with ethanol (−) or R1881 (+) (B) were infected with lentivirus expressing Scramble, AR, or ETV1 shRNA, Western blotting was performed and quantified using ImageJ for AR, ETV1, and Twist1 proteins, which are shown as bar graphs (normalized to β-actin). Bar graphs represent averages of 3 independent experiments plus standard deviations. The Student’s *T*-test was performed to show statistical significance (*p < 0.05, ***p < 0.01), as indicated by the asterisks.

10.7717/peerj.8921/supp-5 Supplemental Information 5 Coactivators have differential activities on the ETV1 activation of the Twist1 promoter. (A and B) HEK cells were co-transfected with Twist1-Luc and ETV1 expression plasmid (+) or empty plasmid (−), as indicated. Expression plasmids for coactivators SRC-1, SRC-2, SRC-3, p300, or CBP were also co-transfected with ETV1, as indicated. Bar graphs represent average Luciferase activities of three independent experiments plus standard deviations. The Student’s *T*-test was performed to show statistical significance (*p < 0.05, ***p < 0.01), as indicated by the asterisks.

10.7717/peerj.8921/supp-6 Supplemental Information 6 Knockdown of Twist1 does not affect growth of prostate cancer cells. (A and B) LNCaP cells were transfected with control (Ctrl), AR, or Twist1 siRNA and measured for cell number using the MTT assay. In B, cells grown in 2% DCC-serum were treated with ethanol (−) or 10 nM R1881 (+). Bar graphs represent averages of 3 independent experiments plus standard deviations. The Student’s *T*-test was performed to show statistical significance (*p < 0.05, ***p < 0.01), as indicated by the asterisks.

10.7717/peerj.8921/supp-7 Supplemental Information 7 Dataset for Fig. 1 and Figs. S1 and S2.

10.7717/peerj.8921/supp-8 Supplemental Information 8 Dataset for Fig. S3.

10.7717/peerj.8921/supp-9 Supplemental Information 9 Dataset for Fig. 2 and Fig. S4.

10.7717/peerj.8921/supp-10 Supplemental Information 10 Dataset for Fig. 3 and Fig. S5.

10.7717/peerj.8921/supp-11 Supplemental Information 11 Dataset for Fig. 4 and Fig. S6.

10.7717/peerj.8921/supp-12 Supplemental Information 12 Raw Western for Fig. 1A.

10.7717/peerj.8921/supp-13 Supplemental Information 13 Raw Western for Fig. 1A2.

10.7717/peerj.8921/supp-14 Supplemental Information 14 Raw Western for Fig. 1A3.

10.7717/peerj.8921/supp-15 Supplemental Information 15 Raw Western for Fig. 1B.

10.7717/peerj.8921/supp-16 Supplemental Information 16 Raw Western for Fig. 1C.

10.7717/peerj.8921/supp-17 Supplemental Information 17 Raw Western for Fig. 2D.

10.7717/peerj.8921/supp-18 Supplemental Information 18 Raw Western for Fig. 2E.

10.7717/peerj.8921/supp-19 Supplemental Information 19 Raw data for Fig. 4G and 4H.

## Additional Information and Declarations

### Data Availability

The following information was supplied regarding data availability: The raw data (images of X-ray films of all Western blots that were used to generate all Western blots found on the figures of the article) are available as Supplemental Files.
