# Supplementary figures and images for: Androgen up-regulation of Twist1 gene expression is mediated by ETV1
Source: PeerJ. 2020 Apr 9;8:e8921. doi: 10.7717/peerj.8921 (PMC7151753; doi:10.7717/peerj.8921)

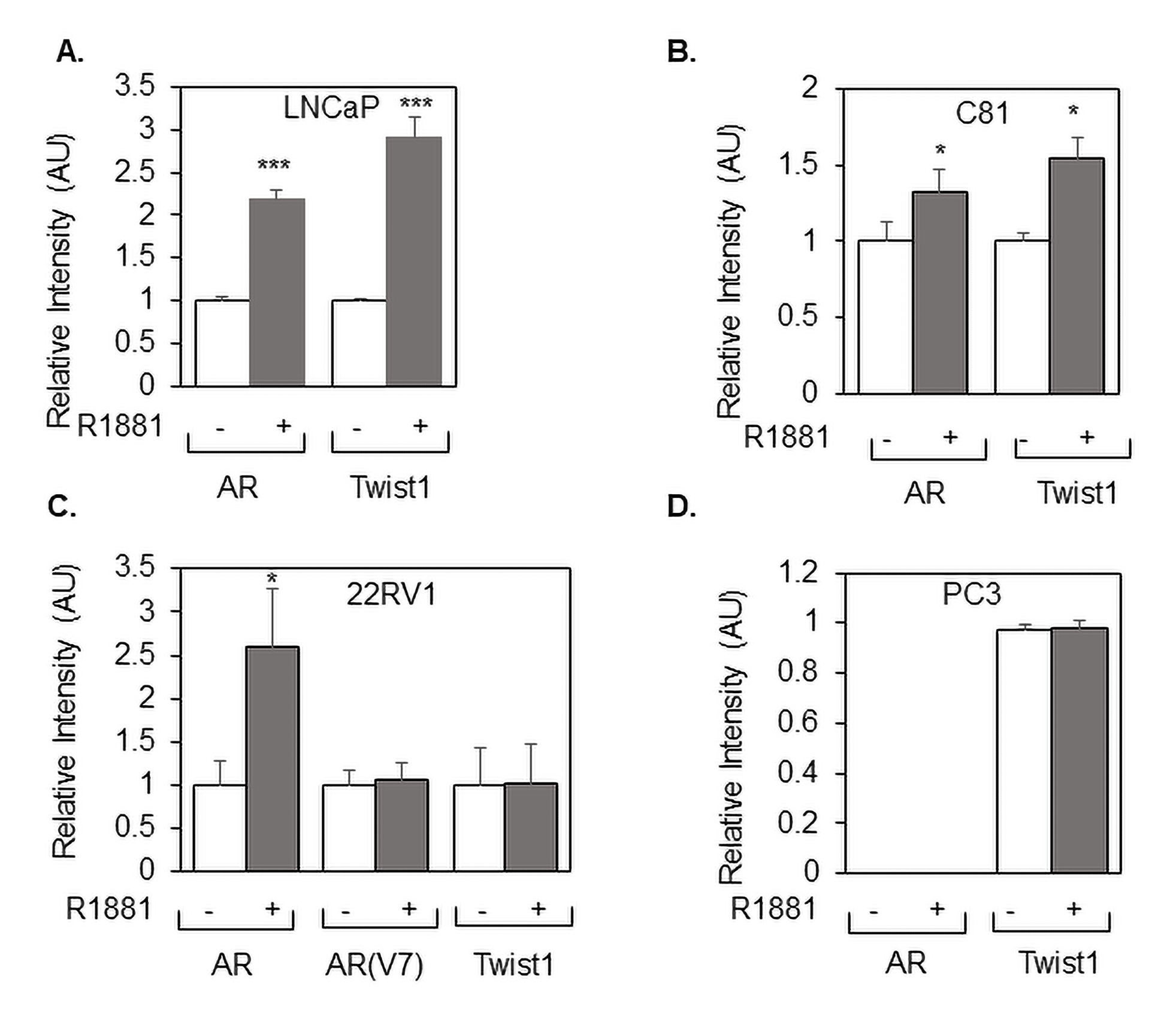

Supplement: Supplemental Information 1 — LNCaP (A), C81 (B), CWR-22Rv1 (C), or PC3 (D) cells were grown in 2% DCC-serum with ethanol (−) or 10 nM R1881 (+) for 48 h, Western blotting was performed and quantified using ImageJ for AR and Twist1 proteins, which are shown as bar graphs (normalized to β-actin). Bar graphs represent averages of three independent experiments plus standard deviations. The Student’s T-test was performed to show statistical significance (*p < 0.05, ***p < 0.01), as indicated by the asterisks. [file peerj-08-8921-s001.png]

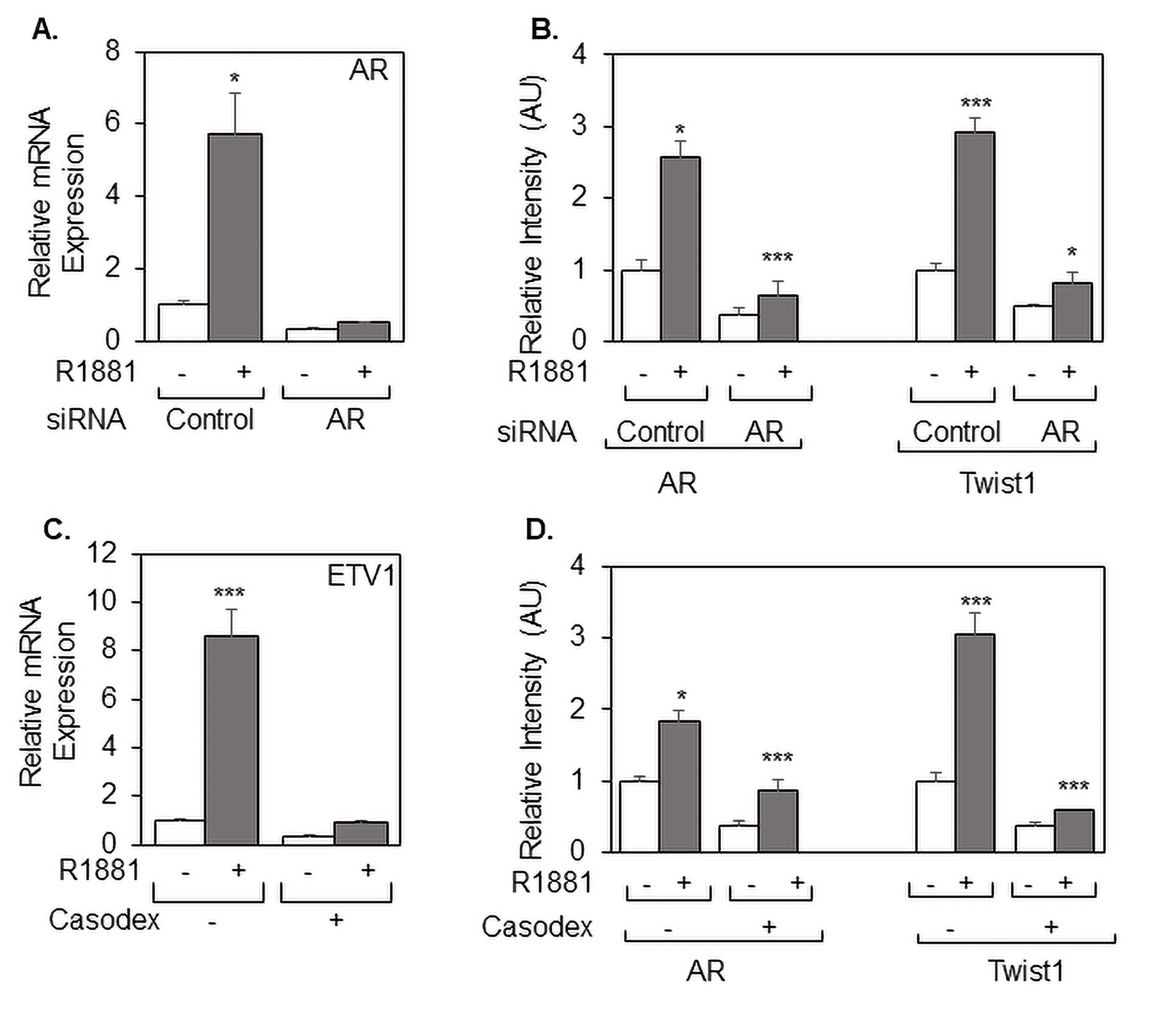

Supplement: Supplemental Information 2 — LNCaP cells were transfected with control or AR siRNA (A and B) or treated with 50 nM Casodex (an anti-androgen) (C and D) in the presence of ethanol (−) or 10 nM R1881 (+) and expression of AR (A), or ETV1 (C) were measured using qRT-PCR. The relative band intensity of AR and Twist1 proteins were quantified using ImageJ and shown as bar graphs (normalized to β-actin) in (B) and (D). Bar graphs represent averages of three independent experiments plus standard deviations. The Student’s T-test was performed to show statistical significance (*p < 0.05, ***p < 0.01), as indicated by the asterisks. [file peerj-08-8921-s002.png]

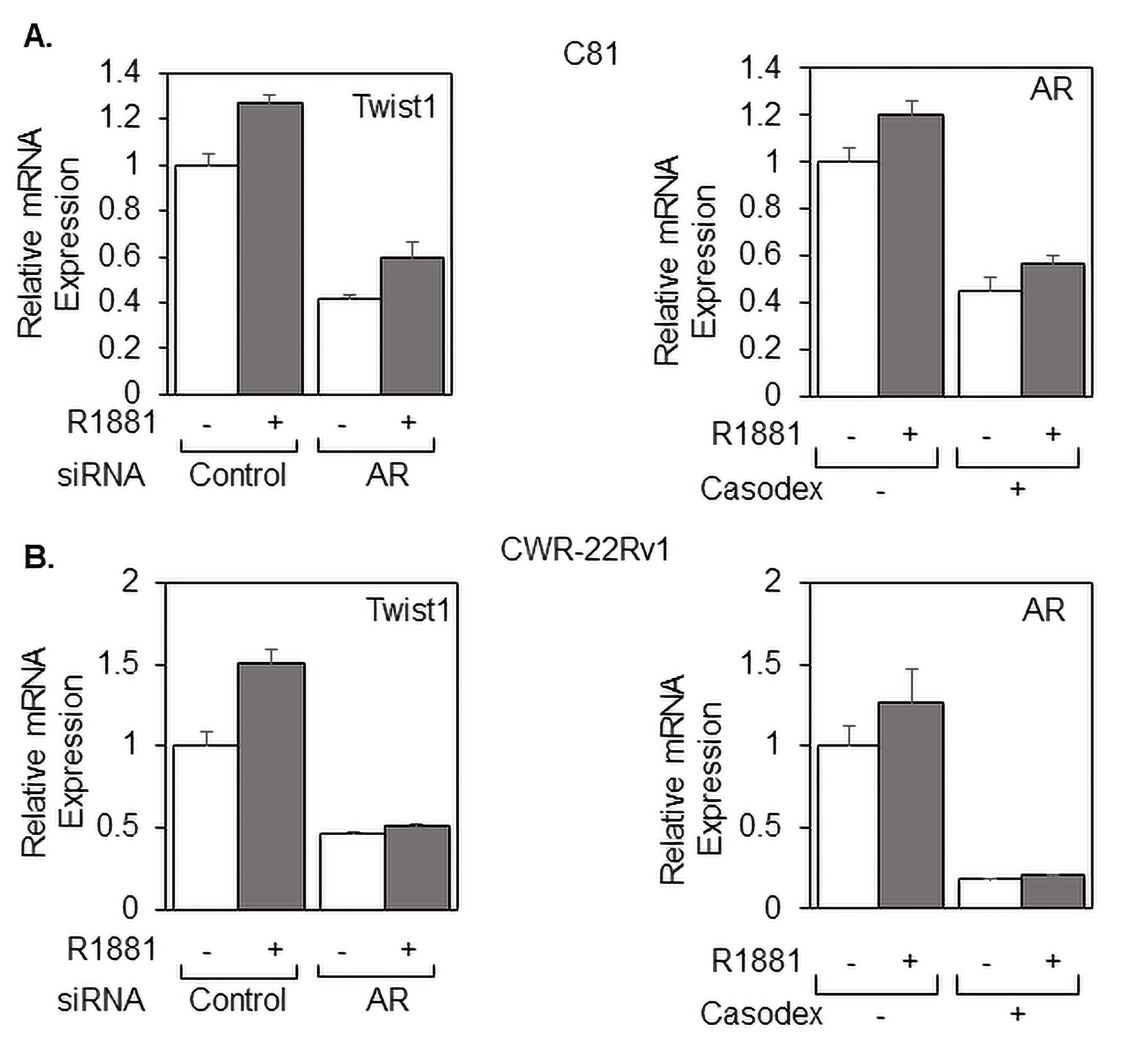

Supplement: Supplemental Information 3 — (A) C81 or (B) CWR-22Rv1 cells grown in 2% DCC with ethanol (−) or 10 nM R1881 (+) were transfected with control or AR siRNA and relative gene expression of Twist1 and AR were measured by qRT-PCR. Bar graphs represent average of 3 independent experiments plus standard deviations. The Student’s T-test was performed to show statistical significance (*p < 0.05, ***p < 0.01), as indicated by the asterisks. [file peerj-08-8921-s003.png]

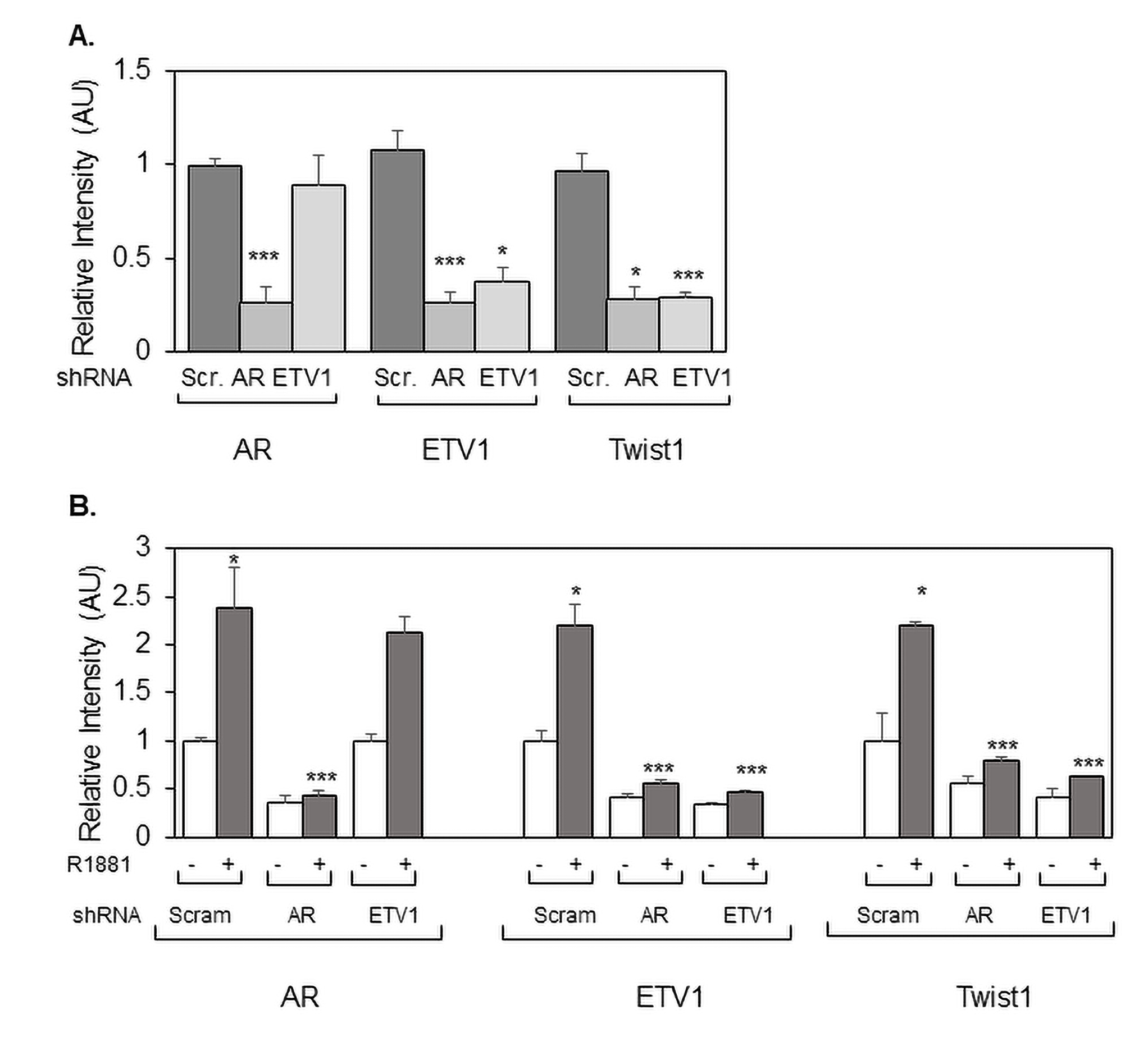

Supplement: Supplemental Information 4 — LNCaP cells grown in full serum (A) or treated with ethanol (−) or R1881 (+) (B) were infected with lentivirus expressing Scramble, AR, or ETV1 shRNA, Western blotting was performed and quantified using ImageJ for AR, ETV1, and Twist1 proteins, which are shown as bar graphs (normalized to β-actin). Bar graphs represent averages of 3 independent experiments plus standard deviations. The Student’s T-test was performed to show statistical significance (*p < 0.05, ***p < 0.01), as indicated by the asterisks. [file peerj-08-8921-s004.png]

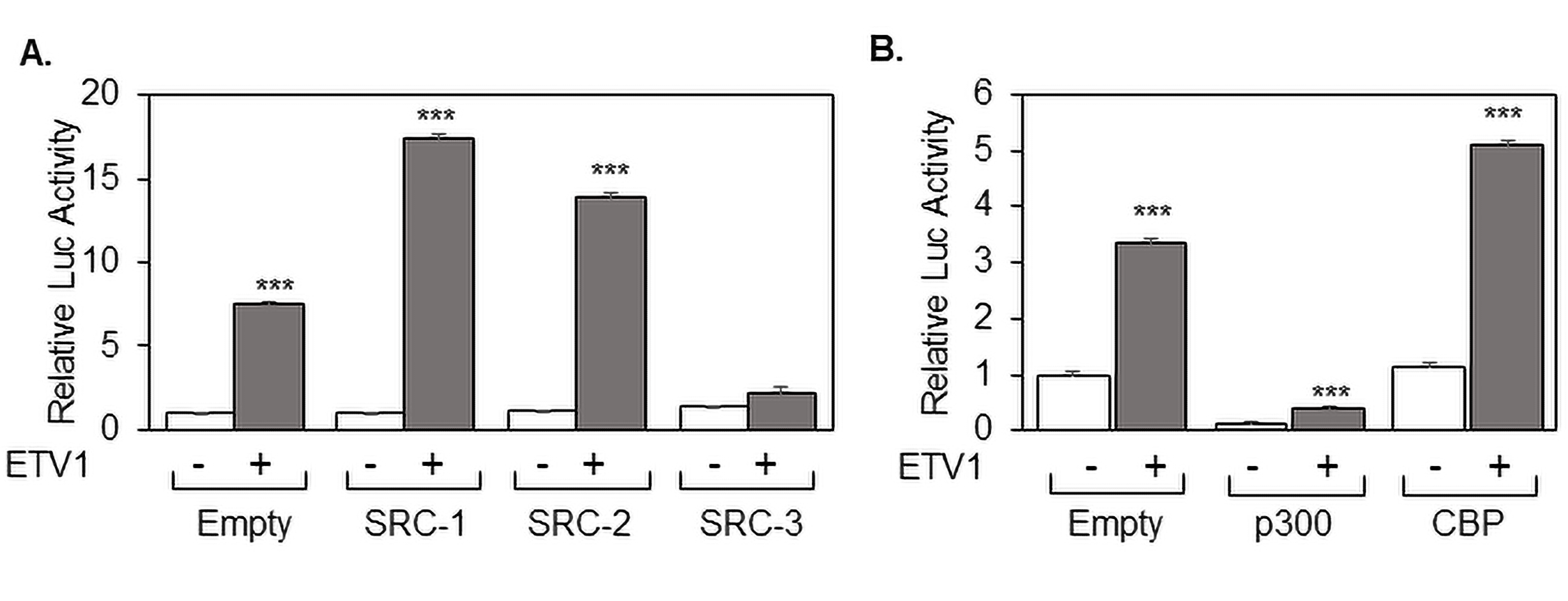

Supplement: Supplemental Information 5 — (A and B) HEK cells were co-transfected with Twist1-Luc and ETV1 expression plasmid (+) or empty plasmid (−), as indicated. Expression plasmids for coactivators SRC-1, SRC-2, SRC-3, p300, or CBP were also co-transfected with ETV1, as indicated. Bar graphs represent average Luciferase activities of three independent experiments plus standard deviations. The Student’s T-test was performed to show statistical significance (*p < 0.05, ***p < 0.01), as indicated by the asterisks. [file peerj-08-8921-s005.png]

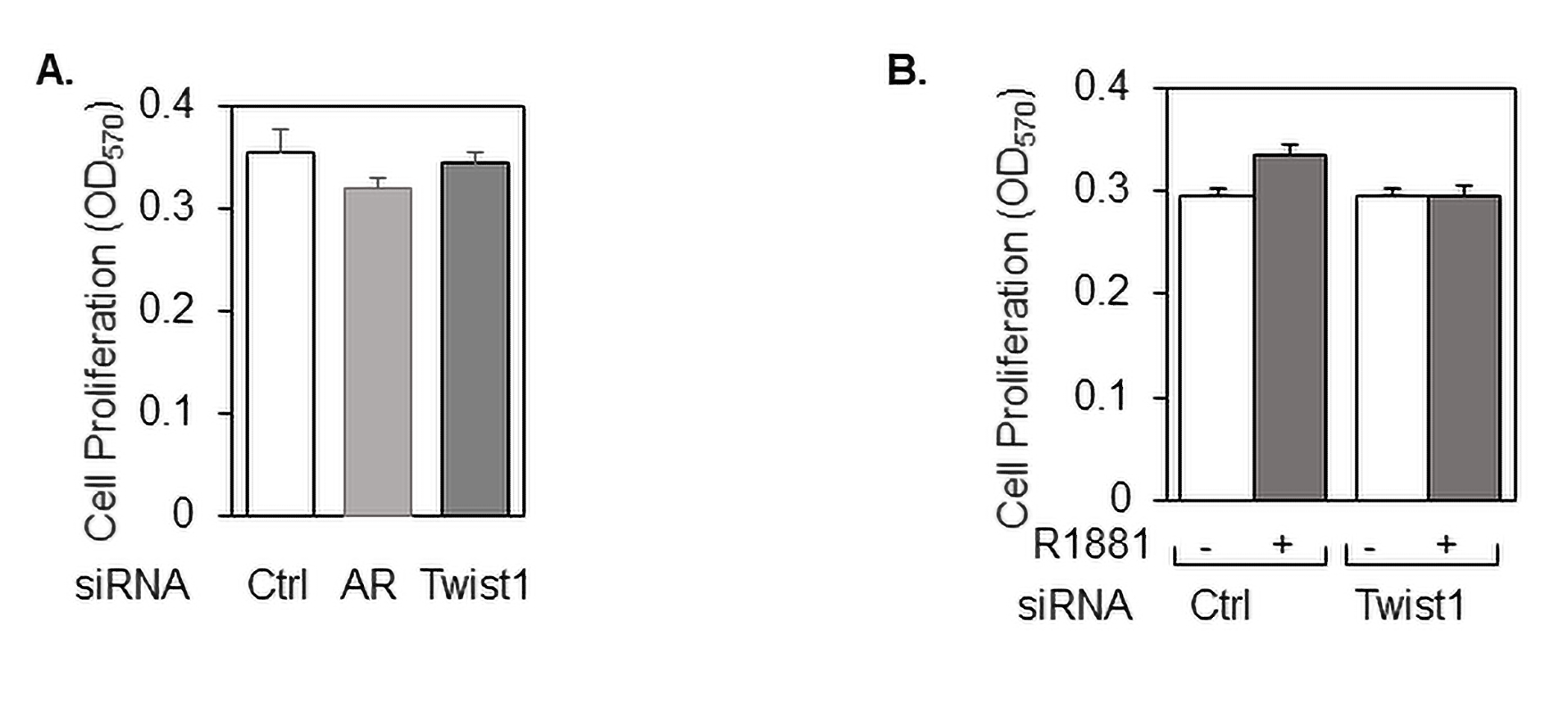

Supplement: Supplemental Information 6 — (A and B) LNCaP cells were transfected with control (Ctrl), AR, or Twist1 siRNA and measured for cell number using the MTT assay. In B, cells grown in 2% DCC-serum were treated with ethanol (−) or 10 nM R1881 (+). Bar graphs represent averages of 3 independent experiments plus standard deviations. The Student’s T-test was performed to show statistical significance (*p < 0.05, ***p < 0.01), as indicated by the asterisks. [file peerj-08-8921-s006.png]

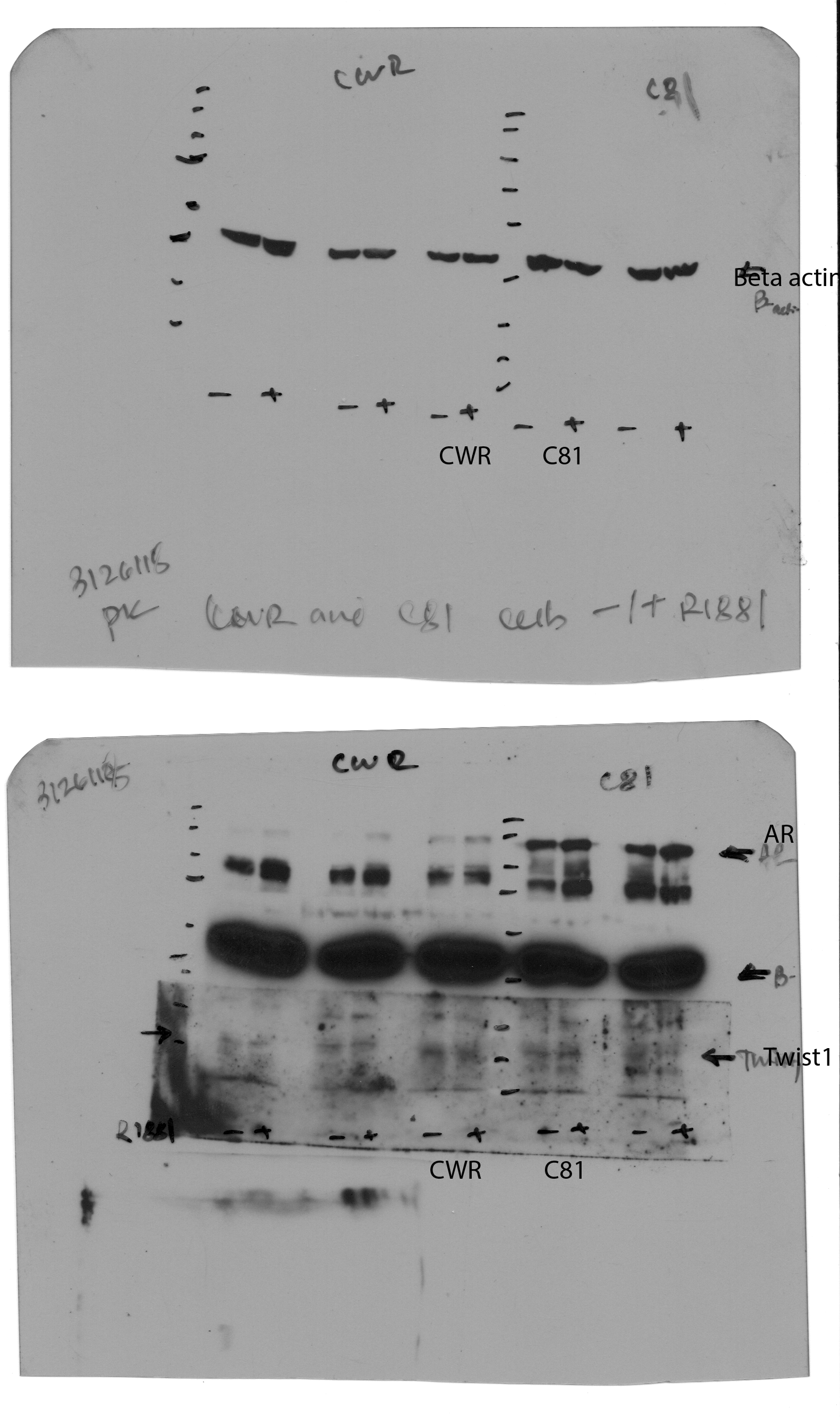

Supplement: Supplemental Information 12 [file peerj-08-8921-s012.tif]

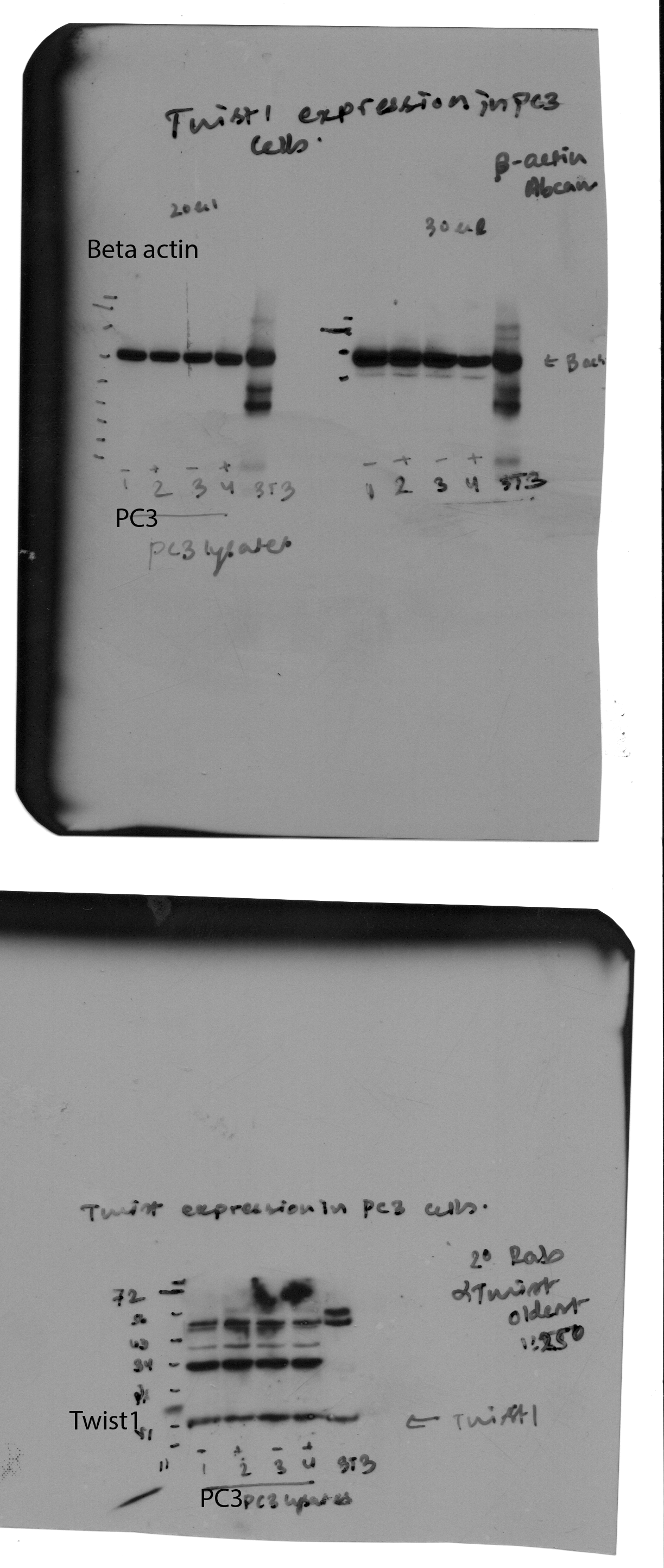

Supplement: Supplemental Information 13 [file peerj-08-8921-s013.tif]

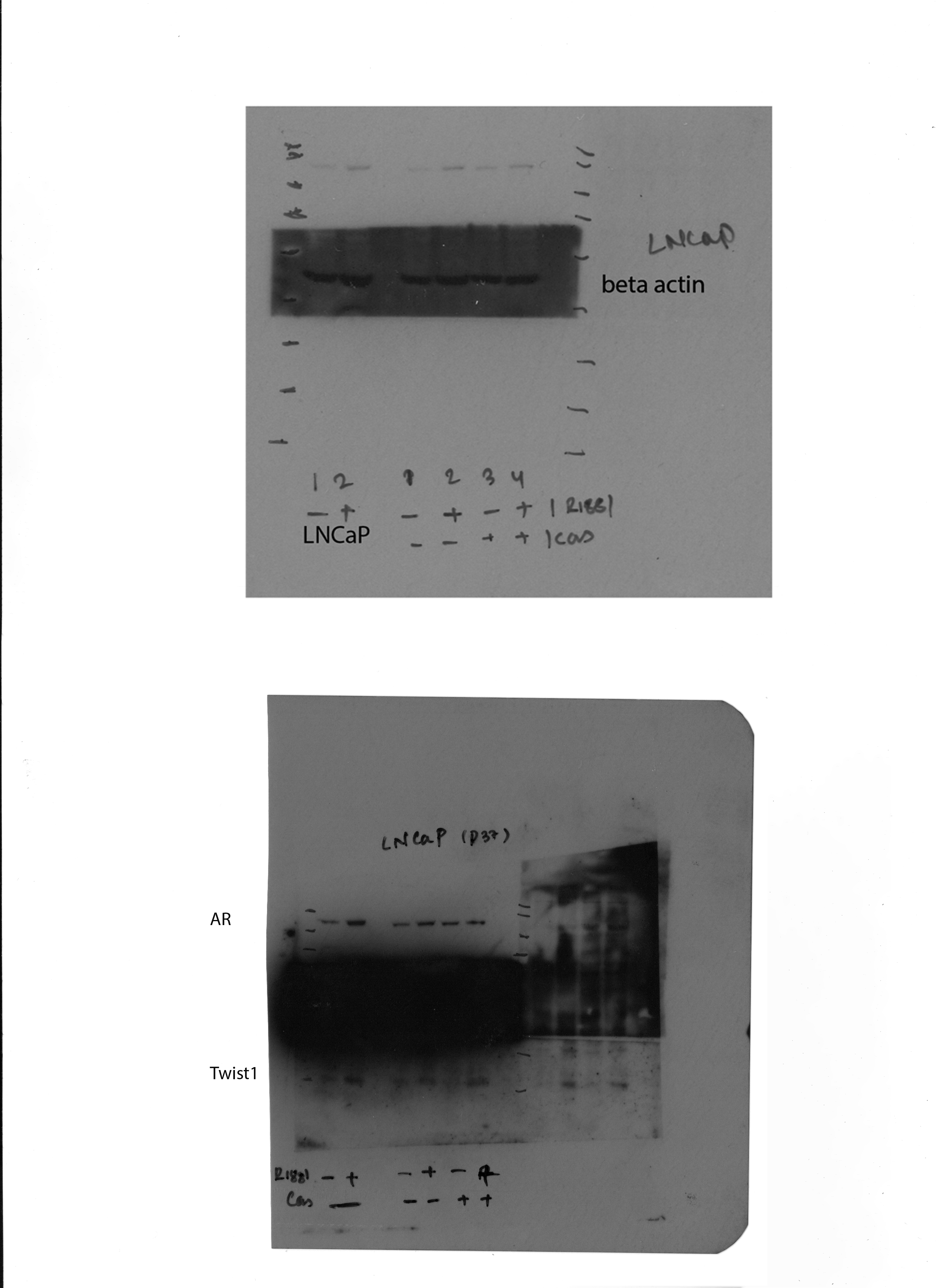

Supplement: Supplemental Information 14 [file peerj-08-8921-s014.tif]

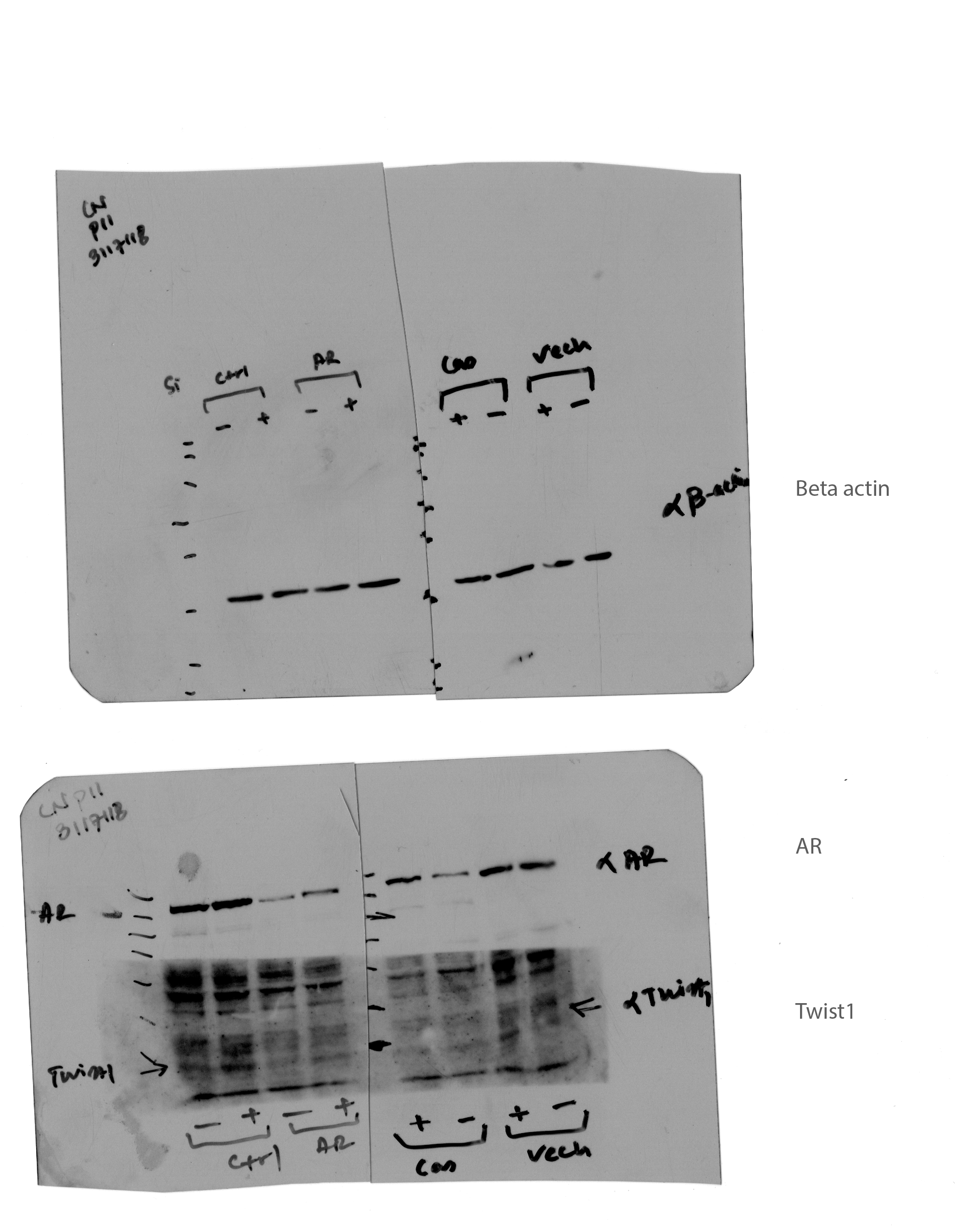

Supplement: Supplemental Information 15 [file peerj-08-8921-s015.tif]

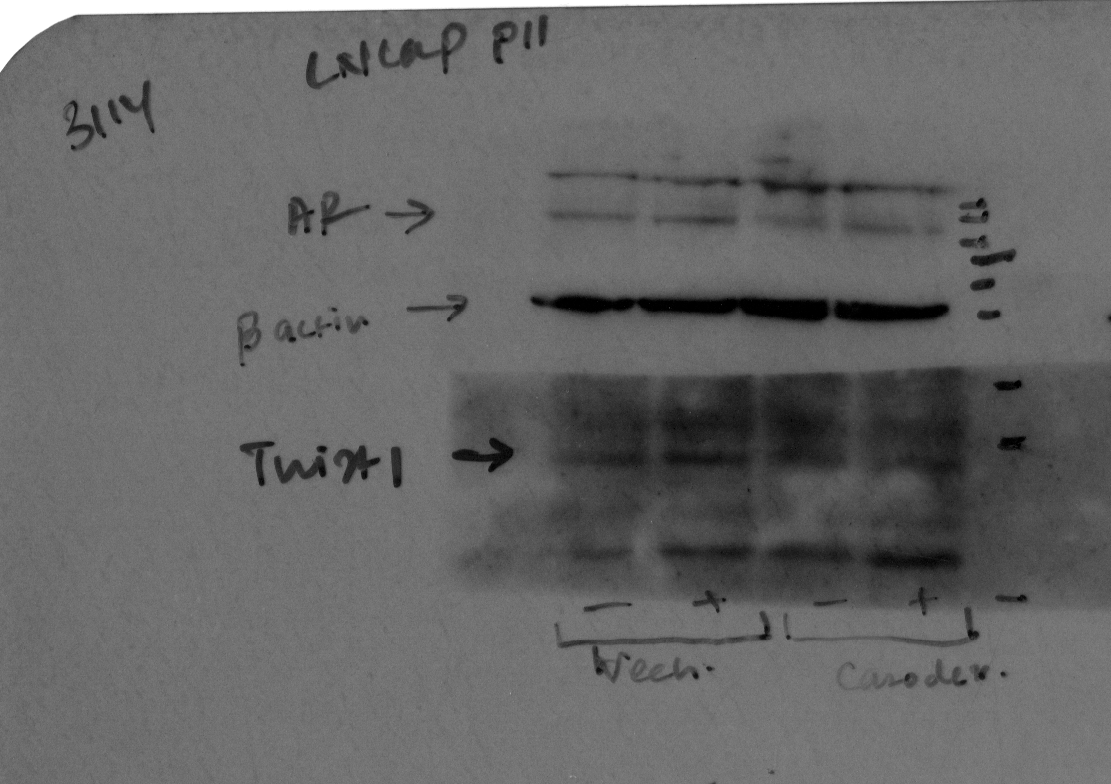

Supplement: Supplemental Information 16 [file peerj-08-8921-s016.tif]

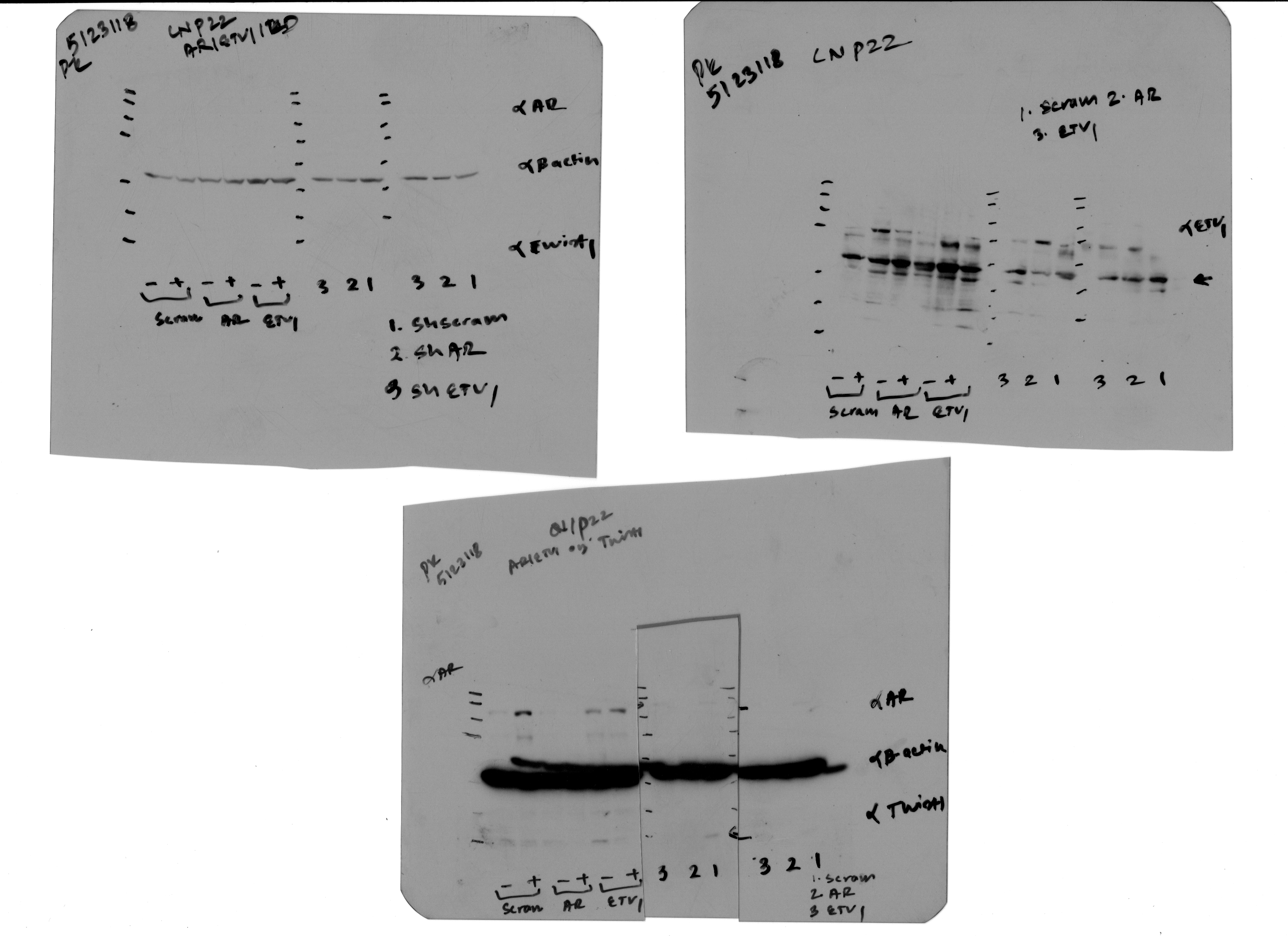

Supplement: Supplemental Information 17 [file peerj-08-8921-s017.tif]

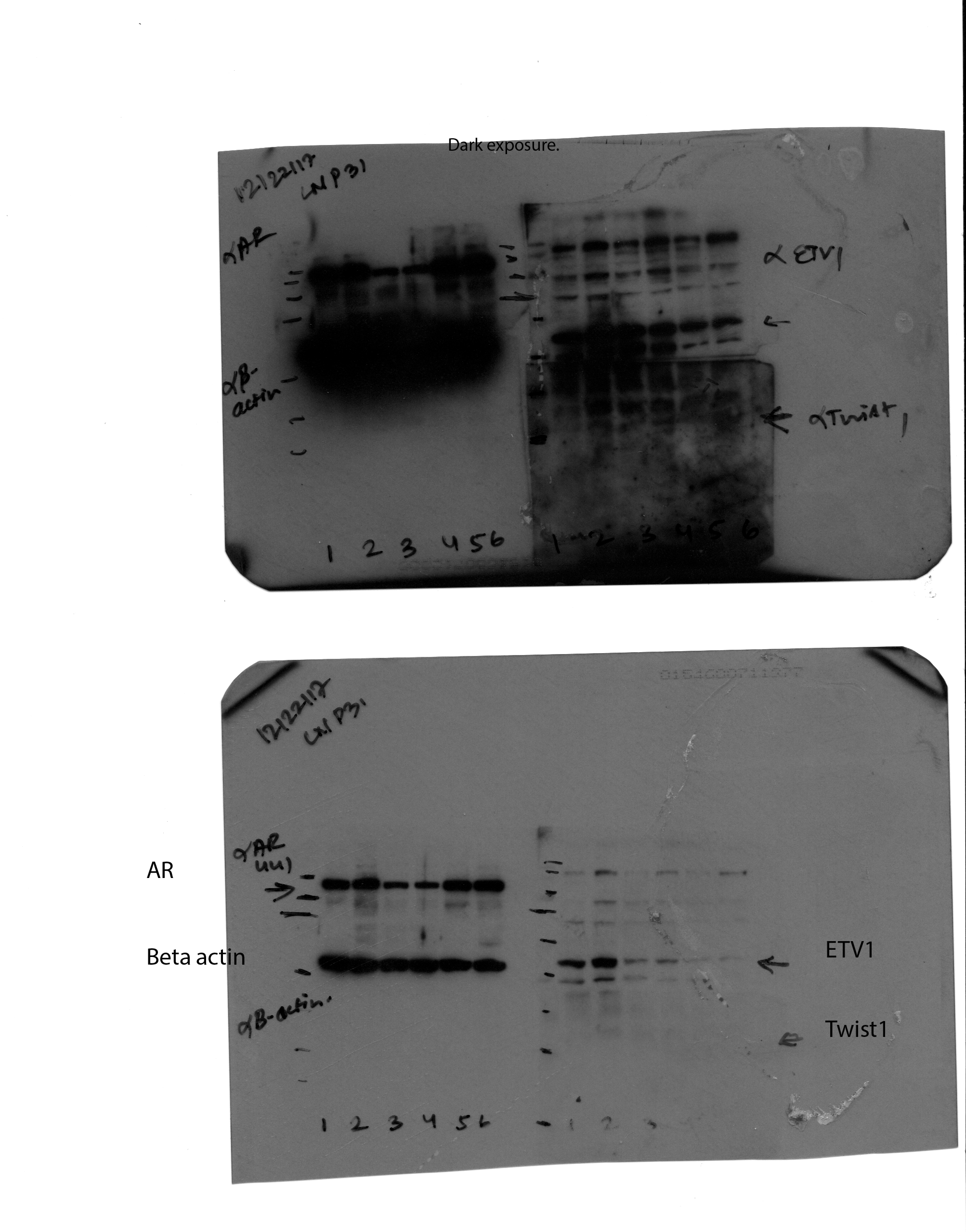

Supplement: Supplemental Information 18 [file peerj-08-8921-s018.tif]

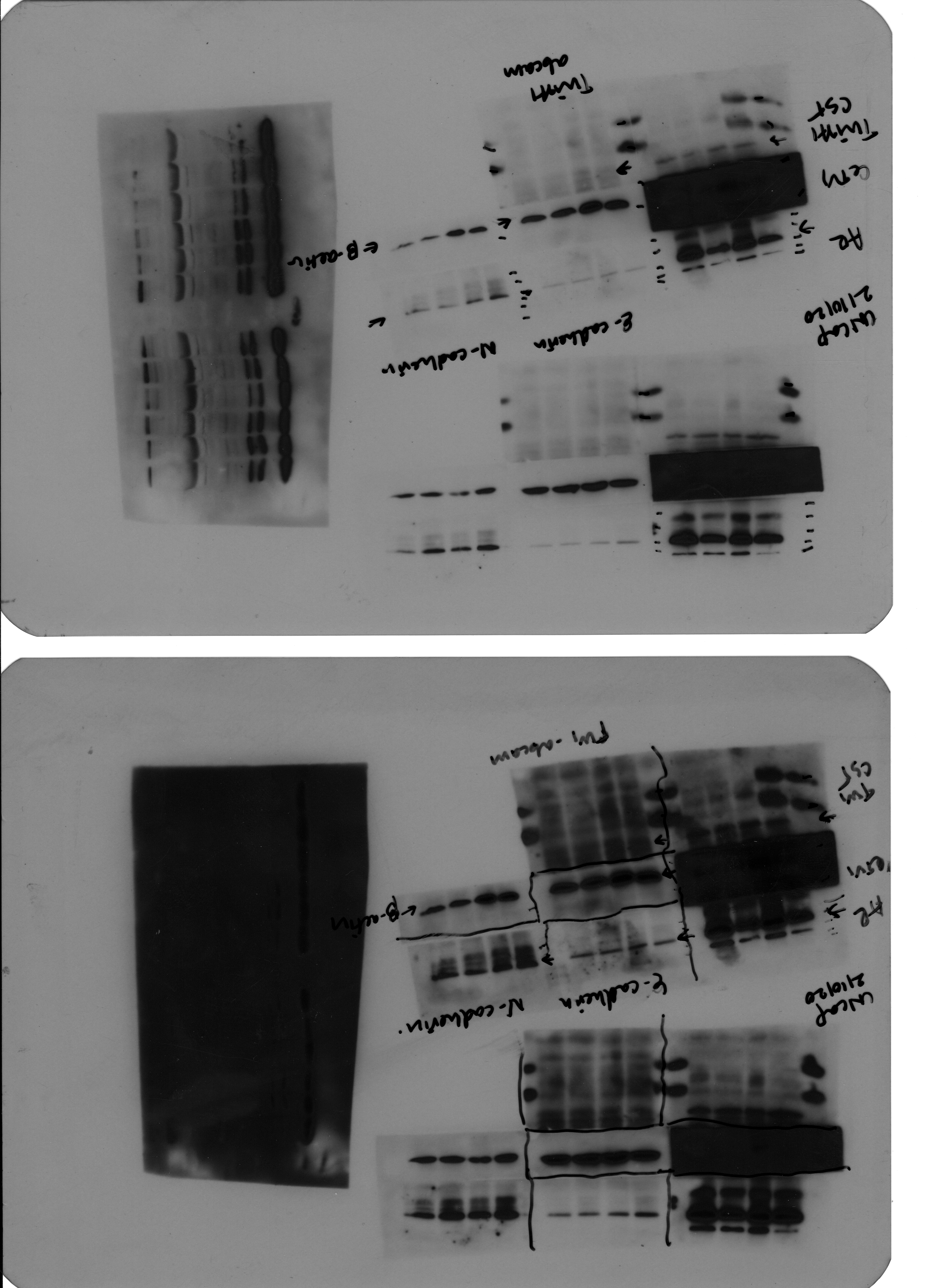

Supplement: Supplemental Information 19 [file peerj-08-8921-s019.tif]
